# Characterization of putative DD-carboxypeptidase-encoding genes in *Mycobacterium smegmatis*

**DOI:** 10.1038/s41598-019-41001-x

**Published:** 2019-03-26

**Authors:** Christopher S. Ealand, Rukaya Asmal, Lethabo Mashigo, Lisa Campbell, Bavesh D. Kana

**Affiliations:** 1 DST/NRF Centre of Excellence for Biomedical TB Research, School of Pathology, Faculty of Health Sciences, University of the Witwatersrand and the National Health Laboratory Service, P.O. Box 1038, Johannesburg, 2000 South Africa; 2 MRC-CAPRISA HIV-TB Pathogenesis and Treatment Research Unit, Centre for the AIDS Programme of Research in South Africa, CAPRISA, Durban, South Africa

## Abstract

Penicillin binding proteins (PBPs) are the target of numerous antimicrobial agents that disrupt bacterial cell wall synthesis. In mycobacteria, cell elongation occurs through insertion of nascent cell wall material in the sub-polar region, a process largely driven by High Molecular Weight PBPs. In contrast, the function of DD-carboxypeptidases (DD-CPases), which are Low Molecular Weight Class 1C PBPs, in mycobacteria remains poorly understood. *Mycobacterium smegmatis* encodes four putative DD-CPase homologues, which display homology to counterparts in *Escherichia coli*. Herein, we demonstrate that these are expressed in varying abundance during growth. Deletion of MSMEG_1661, MSMEG_2433 or MSMEG_2432, individually resulted in no defects in growth, cell morphology, drug susceptibility or spatial incorporation of new peptidoglycan. In contrast, deletion of MSMEG_6113 (*dacB*) was only possible in a merodiploid strain expressing the homologous *M. tuberculosis* operon encoding Rv3627c (*dacB*), Rv3626c, Rv3625c (*mesJ*) and Rv3624c (*hpt*), suggestive of essentiality. To investigate the role of this operon in mycobacterial growth, we depleted gene expression using anhydrotetracycline-responsive repressors and noted reduced bipolar peptidoglycan synthesis. These data point to a possible role for this four gene operon, which is highly conserved across all mycobacterial species, in regulating spatial localization of peptidoglycan synthesis.

## Introduction

Penicillin binding proteins (PBPs) are a group of evolutionary related bacterial proteins that have formed the basis of successful antibiotic chemotherapy for decades due to their vulnerability to β-lactam antibiotics. These proteins are ubiquitous in peptidoglycan (PG)-containing bacteria and can be classified into low molecular weight (LMW) or high molecular weight (HMW) counterparts, both playing important roles in PG cross-linking^1–4^. PG is a three-dimensional, cross-linked lattice-like structure surrounding bacterial cells that allows for maintenance of cytosolic turgor pressure and cell shape. Lipid-linked, nascent PG monomers are synthesized in the bacterial cytoplasm, followed by translocation into the periplasmic compartment where they are incorporated into the existing cell wall by PBPs^2,4^. In most cases, PBPs are acyl-serine transferases that retain either mono- or bi-functional catalytic proficiencies, comprised of either individual, or combinations of transglycosylase, transpeptidase, DD-Carboxypeptidase (DD-CPase), LD-carboxypeptidase or endopeptidase activities^2,5^.

Studies in *Escherichia coli* have identified an extensive complement of HMW and LMW PBPs where the latter, whilst being expressed in greater abundance, are non-essential for growth in axenic culture^6–10^. The majority of *E. coli* LMW PBPs are DD-CPases and/or endopeptidases and some of these, such as PBP5 (DacA), play a notable role in maintenance of cell diameter, overall lateral contour and surface topological features^11–13^. In mutants lacking up to seven PBPs, those cells still expressing PBP5 remained viable and retained normal morphology. Combinatorial deletion of up to eight HMW and LMW PBPs in *E. coli* resulted in several mutants defective for PBP5 in combination with other PBPs wherein morphological defects, enhanced sensitivity to antibiotics and increased cell lysis were observed^7^. *E. coli* PBP5 and other LMW PBPs assist in coordination of septal placement of FtsZ and loss of LMW PBPs resulted in asymmetric cell division and branching of cells^14^. In *E. coli* and other bacterial species such as *Bacillus subtilis* and *Listeria monocytogenes*, deletion of the PBP5 homologue affected PG maturation, leading to decreased levels of tripeptide side-chains and increased levels of pentapeptide side-chains^5,15–17^. In addition, both DacB (PBP5*) and DacF are involved in sporulation either through regulation of spore cortex synthesis or modification of PG composition^18–20^. Similarly, DacF and DacB have been implicated in spore cortex synthesis and heat resistance of spores in *Clostridium perfringens*^21,22^. The role of DD-CPases in non-replicative states is further highlighted in the generation of aberrantly shaped cells during the transition from a replicative to a viable but non-culturable (VBNC) state in *Vibrio parahaemolyticus*^23^.

Relative to well-characterized bacterial species, the role of LMW PBPs in mycobacterial growth, cell expansion and modulation of the PG structure requires further exploration. In a previous study from our group, we identified a multiplicity of LMW PBPs in both pathogenic and non-pathogenic mycobacteria pointing to a potentially important role for these proteins^24^. This notion is supported by several studies that demonstrated physiological roles for LMW PBPs: (i) Over-expression of Rv2911 (*dacB2*) from *Mycobacterium tuberculosis*, in the non-pathogenic saprophyte, *Mycobacterium smegmatis*, resulted in reduced growth, altered colony morphology, defective sliding motility and decreased biofilm formation^25^; (ii) deletion of *dacB2* from *M. tuberculosis* inhibited growth in minimal media under acidic conditions, and also resulted in enhanced survival in THP-1 human macrophage-like cells^25^; (iii) the β-lactam antibiotic, meropenem, directly inhibited DacB2 through covalent linkage with the enzyme which resulted in polar swelling, eventually leading to cell lysis^26^ and (iv) expression of an *M. smegmatis* LMW PBP with DD-CPase and beta-lactamase activity, MSMEG_2433, corrected morphological defects in a septuple PBP mutant of *E. coli*^27^. These studies suggest that mycobacterial DD-CPases function similarly to the *E. coli* homologues in regulating cross-linking and maintaining cell shape. In this study, we attempt to expand the current understanding of DD-CPase function in mycobacterial PG synthesis by constructing and characterizing mutant and knockdown strains defective for putative DD-CPase-encoding genes in *M. smegmatis*.

## Results

### Bioinformatics analysis of putative DD-CPase homologues in mycobacterial species

**Figure 1:**
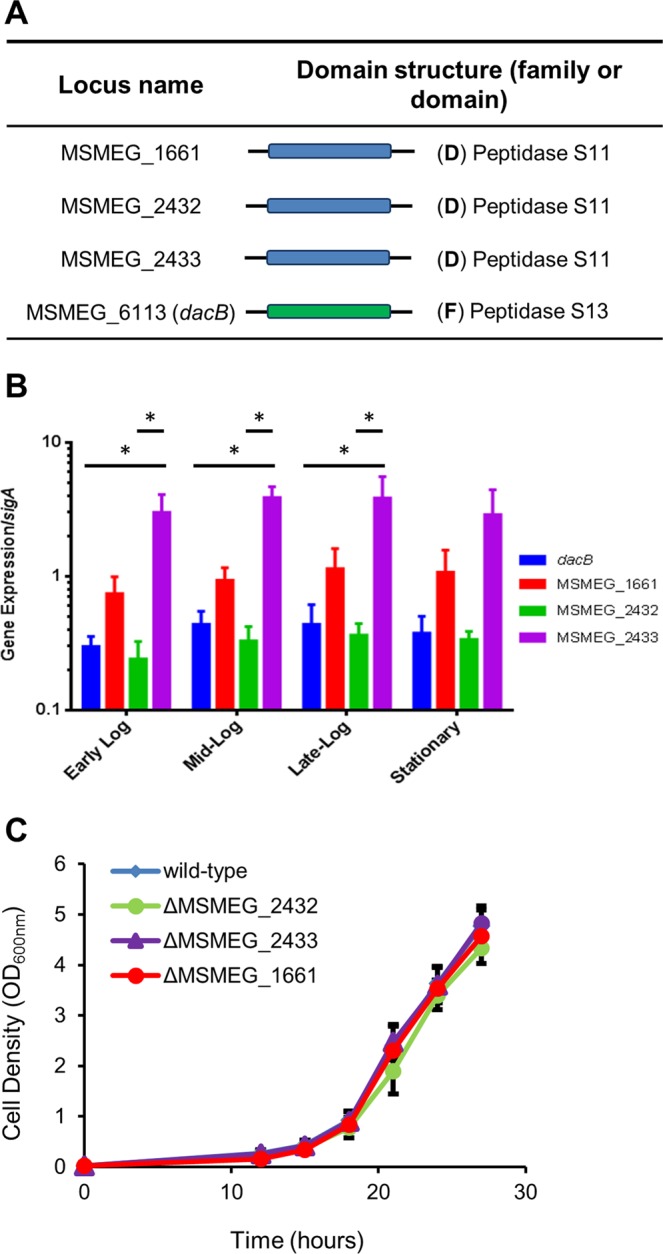
LMW PBP homologues, with DD-CPase activity, in *M. smegmatis*. (**A**) Gene locus and domain architecture of DD-CPases. Data were accessed in January 2018. Proteins were identified by BLASTp (https://blast.ncbi.nlm.nih.gov/Blast.cgi) and KEGG (www.genome.jp), using *E. coli* homologues as query sequences. Gene names are represented as annotations used in KEGG for standardization. Domain (D) or family (F) annotation was obtained at InterPro (https://www.ebi.ac.uk/interpro/): Blue – peptidase_S11 and green – PF0794. Schematic diagrams are not drawn to scale. (**B**) Gene expression at various points during growth phase. Transcripts were normalized to *sigA*. ^*^Represents a statistically significant difference for p < 0.05. (**C**) Single gene deletion mutants were not impaired for growth and were deemed non-essential. MSMEG_6113 (*dacB*) could not be deleted from the genome.

Protein sequences corresponding to *E. coli* LMW PBP homologues were used in a BLAST analysis (https://blast.ncbi.nlm.nih.gov/Blast.cgi) to identify mycobacterial homologues. The genome of *M. smegmatis* mc^2^155 carries four putative DD-CPase-encoding genes including MSMEG_2433, MSMEG_2432, MSMEG_1661, and MSMEG_6113 (annotated as *dacB*). *M. tuberculosis* H37Rv encodes three DD-CPase homologues including Rv3330 (annotated as *dacB1*), Rv2911 (annotated as *dacB2*) and Rv3627c (direct homolog of *dacB* in *M. smegmatis*)^24^. Consistent with the *E. coli* homologues^5^, amino acid alignments revealed a conservation of PBP domains and residues required for DD-CPase activity, i.e. S*xx*K, S*x*N and KTG in all mycobacterial homologues, with the exception of MSMEG_6113 (DacB) and Rv3627c (Supplementary Fig. S1A,B). The low level of similarity between the *E. coli* DacA protein and MSMEG_6113 (DacB)/Rv3627c is due to the fact these two mycobacterial homologues are longer than the *E. coli* protein, causing misalignment of the critical conserved residues. When MSMEG_6113 (DacB) and Rv3627c are aligned against the other mycobacterial DD-CPase homologues, the aforementioned three critical domains are clearly identifiable in these two proteins (Supplementary Fig. S1B). This, together with the presence of a Peptidase S13 family domain, found in DD-CPases, suggests that both MSMEG_6113 (DacB) and Rv3627c most likely encode putative DD-CPases. The other mycobacterial homologues all contain Peptidase S11 domains (Fig. 1A). Phylogenetic analysis of the mycobacterial DD-CPases revealed that MSMEG_6113 (*dacB*) and Rv3627c are located on a separate branch of their respective phylogenetic trees, suggestive of functional divergence (Supplementary Fig. S2). MSMEG_1900 was identified using BLAST searches with the *E. coli* DacA protein as the query and is annotated as a DD-CPase in SmegmaList (http://svitsrv8.epfl.ch/mycobrowser/smegmalist.html). However, our analysis suggests that it is unlikely to retain DD-CPase activity as all the residues required for acyltransferase activity are absent (Supplementary Fig. S1A). Considering this, we eliminated MSMEG_1900 from further analyses.

### DD-CPase homologues of *M. smegmatis* are expressed in differential abundance during growth

Considering the genetic multiplicity of DD-CPases in *M. smegmatis*, we sought to further interrogate the roles of these proteins during growth. To evaluate the contribution of each, mRNA transcript levels were assessed during early log (optical density [OD]_600nm_ = 0.3), mid-log (OD_600nm_ = 0.7), late log (OD_600nm_ = 2) and stationary (OD_600nm_ = 5) phase in the wild-type strain (mc^2^155). MSMEG_2433 was the most abundant transcript (p < 0.05) followed by MSMEG_1661 (p < 0.05). Differential gene expression between homologues was observed during the different growth phases but the total transcript abundance did not vary significantly across the time-course for each individual gene (Fig. 1B).

### MSMEG_2432, MSMEG_2433 and MSMEG_1661 are dispensable and deletion thereof does not alter growth rate or cellular morphology

To investigate the role of DD-CPases in *M. smegmatis* cellular morphology, we constructed in-frame, unmarked deletions in MSMEG_2432, MSMEG_2433 and MSMEG_1661 using allelic exchange. PCR and southern blot analysis confirmed the genetic integrity of each deletion allele (Supplementary Fig. S3). DD-CPase deficient strains displayed no growth rate defects in broth culture (Fig. 1C). In a recent study, over-expression of the Rv2911 DD-CPase-encoding gene from *M. tuberculosis* in *M. smegmatis* led to abnormal colony morphology^25^. To assess if similar phenotypic effects prevailed in our mutants, colony morphology was assessed. All strains displayed surface cording comparable to the wild-type and no differences in size or color of colonies were observed (Supplementary Fig. S4). Cell morphology in the mutants was also examined using scanning electron microscopy and no gross morphological defects or changes in cell length were detected (Supplementary Fig. S4).

We next sought to determine if DD-CPase deficiency resulted in changes in cell wall or PG stability by using the cell wall-targeting antibiotics ampicillin, vancomycin, ceftriaxone, cefotaxime, cefapirin and D-cylcoserine, as probes. No differences in minimum inhibitory concentrations (MIC) between the mutants and wild-type were observed (data not shown). In addition, no differential increase in susceptibility was observed in the mutant strains in the presence of detergent (0.2% SDS); rather a marginal increase in resistance to SDS was observed in the ΔMSMEG_2433 mutant, but this was not considered to be significant (Supplementary Fig. S5A). In *E. coli*, loss of the LMW PBPs 4, 5 and 7 or combinations thereof, led to defects in biofilm formation^28^ and to test this in our mutant strains, we assessed their ability to form a biofilm at the liquid air interface^29^. Our single DD-CPase deletion mutant strains formed typical floating biofilms that were indistinguishable from the wild-type (Supplementary Fig. S5B). Given the demonstrated role for DD-CPases in removal of the terminal D-Ala residue, we next sought to assess spatial localization of the terminal D-Ala-D-Ala motif found on nascent Lipid II units. For this, we used a BODIPY-vancomycin conjugate, which has previously been used to localize nascent PG subunits in mycobacteria^30,31^. Consistent with previous reports^32–35^, new PG synthesis occurred predominantly at both poles in the wild-type strain. Sub-populations of cells displaying PG synthesis at a single pole or diffused cellular staining were also observed (Supplementary Fig. S6). However, the mutant strains displayed an unaltered PG biosynthesis distribution pattern.

### The MSMEG_6113 (*dacB*) containing gene cluster is essential in *M. smegmatis*

**Figure 2:**
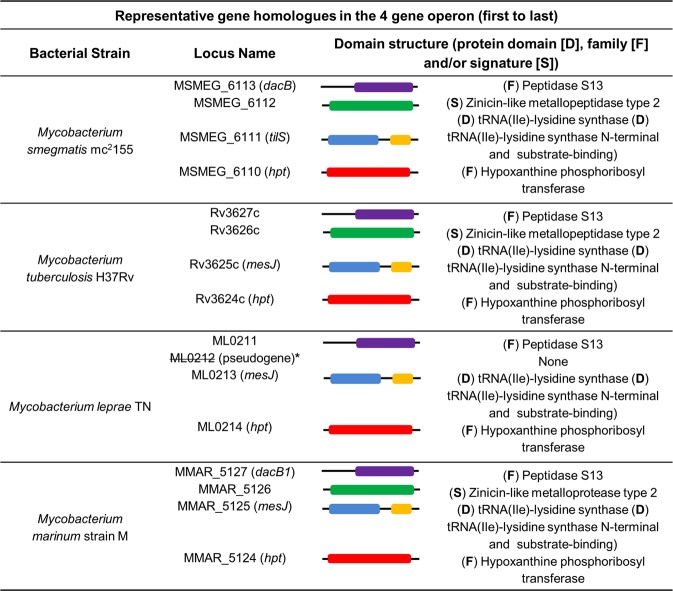
Representative gene homologues representing organization of the “*dacB* operon” in mycobacterial genomes. Data were accessed between January and August 2018. The genomic organization of *M. tuberculosis* homologues was compared to the genomes of the selected mycobacterial species. Protein sequences were retrieved from the Kegg Genome Database (https://www.genome.jp/kegg/genome.html) and interrogated for domain structure (i.e. protein domain [D], family [F] and/or signature [S]) using the InterPro online tool (https://www.ebi.ac.uk/interpro/). Purple, (F) Peptidase S13, D-Ala-D-Ala carboxypeptidase C (IPR000667); green, (F) Zinicin-like metallopeptidase type 2 (IPR018766); blue, (D) IPR011063tRNA(Ile)-lysidine/2-thiocytidine synthase, N-terminal; yellow, (D) IPR015262tRNA(Ile)-lysidine synthase, substrate-binding domain; red, (F) IPR005904 Hypoxanthine phosphoribosyl transferase. *In *M. leprae* TN, a pseudogene of the zinicin-like metalloprotease type 2, ML0212, was identified downstream of the DD-CPase (ML0211) but it lacked protein signatures and could therefore not be classified.

**Figure 3:**
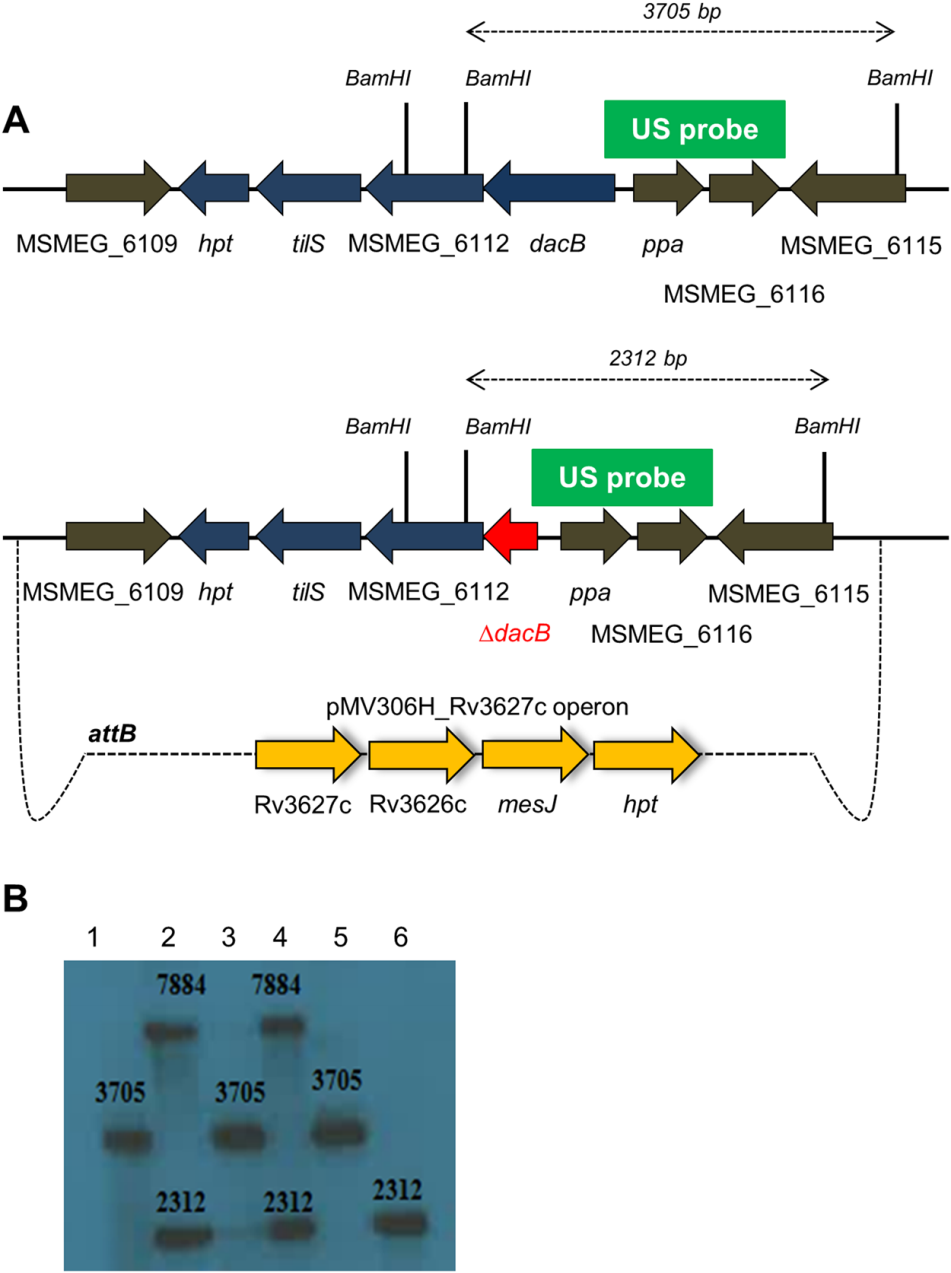
MSMEG_6113 (*dacB*) is essential in *M. smegmatis*. A gene deletion at the native chromosomal site could only be generated in a merodiploid strain containing the homologous *M. tuberculosis* operon at the *attB* site. (**A**) Genomic context of *dacB* operon in wild-type (top). Genomic context of *dacB* operon in ∆*dacB* strain (bottom). A mutant allele could only be generated in the presence of additional copy of the four-gene operon (homologous region from *M. tuberculosis* was cloned into the integrating vector pMV306H). (**B**) Genomic DNA from wild-type, merodiploid ∆*dacB* and potential *dacB* deletion strains were digested with *BamHI* and probed with a DIG-labelled amplicon homologous to a region upstream of *dacB* (green box). Southern hybridization detected the wild-type allele (3705 bp), a single cross-over mutant [SCO] (7884 and 2312) and the mutant allele (2312 bp). Lanes 1–6 correspond to genomic DNA from wild-type, merodiploid and potential *dacB* deletion mutant strains respectively. Lane 6 represents a *dacB* deletion mutant, with the 2312 bp mutant band only.

Our data thus far suggested that three of the four DD-CPase homologues in *M. smegmatis* were dispensable *in vitro*. The direct homologue of the remaining gene, *dacB*, is annotated as essential in *M. tuberculosis*. To confirm this predicted essentiality, we first attempted to delete *dacB* in *M. smegmatis* using a two-step allelic exchange. To confirm the recombination proficiency of the upstream and downstream homologous regions in our knockout vector (p2∆dacBg17), we tested the ability of this plasmid to produce both upstream and downstream single-cross-over (SCO) recombinants, when electroporated into wild-type strain. Southern blot analysis of SCOs revealed the presence of both upstream and downstream SCO strains, confirming that the suicide vector was recombination proficient in both homologous regions (Supplementary Fig. S7A). Representative clones of either SCO were then subjected to sucrose counter selection. A total of 144 colonies were screened and in all cases, counter-selection gave rise to the wild-type allele (data not shown), suggesting that *dacB* is essential. To support this observation, successful deletion of *dacB* at the native site in the chromosome would be dependent on an additional copy of the gene. To test this, we introduced a second copy of *dacB*, with its native promoter sequence, into the upstream SCO strain, at the *attB* phage attachment site, and confirmed the genetic integrity of the strain by Southern blot. Subsequent two-step allelic exchange failed to yield the mutant allele (Supplementary Fig. S7B). A bioinformatics analysis suggested that *dacB* was part of a four-gene operon encoding MSMEG_6113 (*dacB*), MSMEG_6112, *hpt* and *tilS*. The three genes downstream of *dacB* are predicted to encode a zinicin-like metallopeptidase type 2 protein (*tilS*), tRNA(Ile)-lysidine synthase and hypoxanthine phosphoribosyl transferase (*hpt*), respectively (Fig. 2). MSMEG_6112 is a conserved hypothetical protein. Hence, in this operon, only *dacB* is annotated as a protein involved in PG biosynthesis, which is of interest considering that this genetic organization only occurs in mycobacteria (Fig. 2). We reasoned that the inability to delete *dacB*, even in the presence of another copy of the gene, could be the result of downstream polar effects on the remaining genes. To test this, a merodiploid strain containing the entire homologous operon from *M. tuberculosis* (Rv3627c-Rv3626c-*mesJ*-*hpt*), integrated at the *attB* phage attachment site, was constructed (Fig. 3A). In this genetic context, subsequent two-step allelic exchange yielded a mutant *dacB* allele at the native site in the chromosome (Fig. 3B). This confirmed essentiality of the gene cluster.

### Repression of *dacB* gene cluster leads to defects in localization of PG biosynthesis

**Figure 4:**
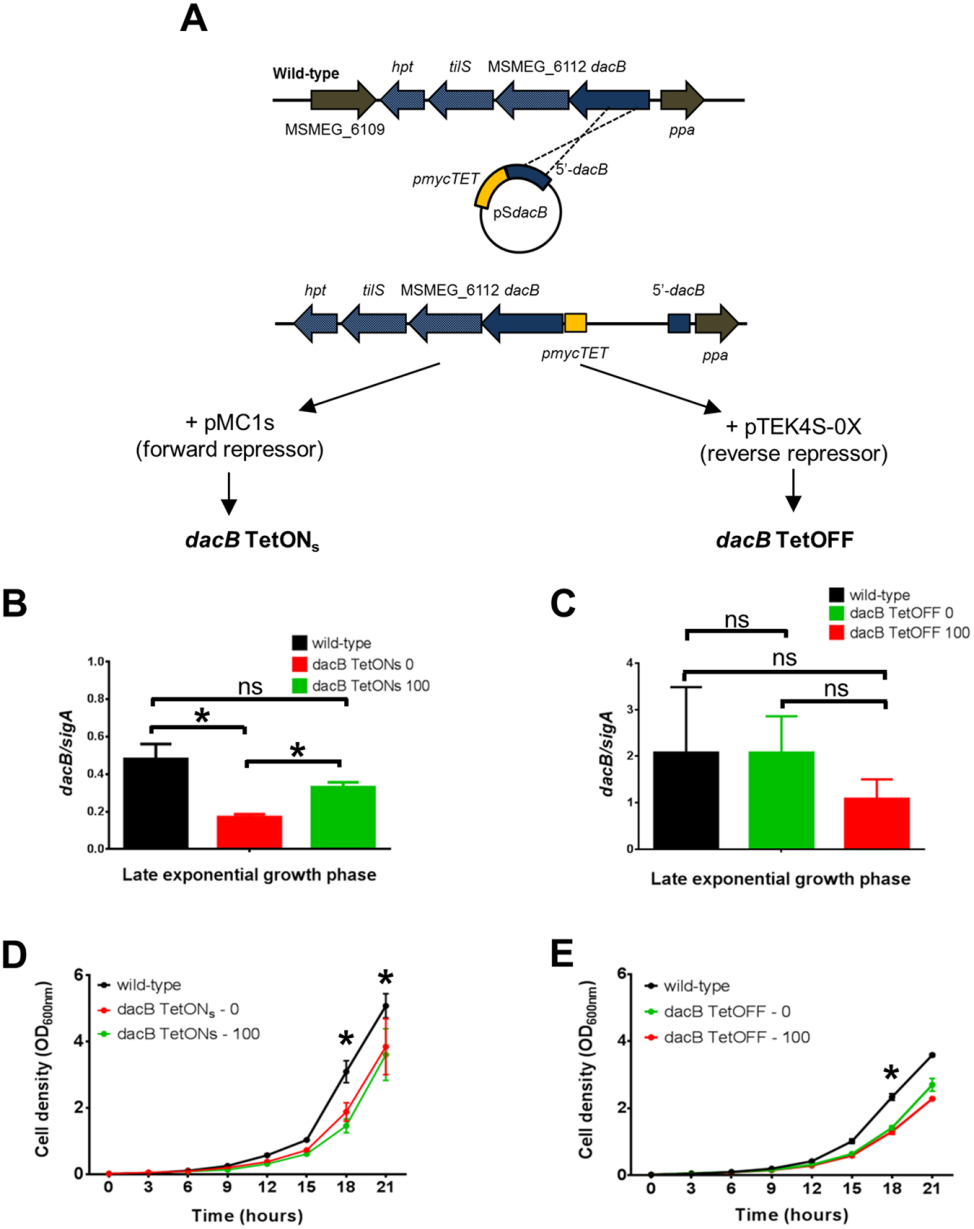
Promoter replacement strategy to assess effects of *dacB* operon repression using two independent anhydrotetracycline (aTc)-responsive repressors. (**A**) The native promoter of the *dacB* operon was replaced with pmycTET (orange box). Repressors (pMC1s, panels on left or pTEK4S-0x, panels on right) were then introduced, independently, into this strain to yield strains *dacB* TetON_s_ and *dacB* TetOFF, respectively. In the absence of aTc, gene expression should be *off* or *on*, respectively. (**B**,**C**) Expression of *dacB* in the absence (0) and presence (100 ng/ml) aTc. Transcript levels, for *dacB*, were measured during late logarithmic growth (18 hours) and normalized against *sigA* levels. (**D**,**E**) Repression of the *dacB* operon (red lines) resulted in a decreased growth rate. Strains were grown in 7H9 supplemented with glucose salts and Tween 80. Samples were removed at 3 hourly intervals to determine optical densities at 600 nm.

As *dacB* could not be individually deleted in *M. smegmatis*, we targeted the operon for transcriptional depletion using promoter replacement with anhydrotetracycline (aTc) inducible systems^36^. For this, we created a strain wherein the tetO operator was introduced upstream of the *dacB* gene cluster, replacing the native promoter (Fig. 4A). The genomic integrity of this strain, designated *dacBTetO*, was confirmed with Southern blotting and PCR (Supplementary Fig. S8). From *dacBTetO*, two strains were created: (i) *dacB* TetON_s_ wherein the ‘forward’ repressor pMC1s was introduced (where supplementation with 0 or 100 ng/ml aTc represented ‘off’ or ‘on’ states, respectively) and (ii) *dacB* TetOFF wherein the ‘reverse’ repressor pTEK4S-0X was introduced (where 0 and 100 ng/ml aTc represented ‘on’ or ‘off’ states, respectively). Thereafter, we monitored *dacB* gene expression as a measure of repression of the gene cluster.

**Figure 5:**
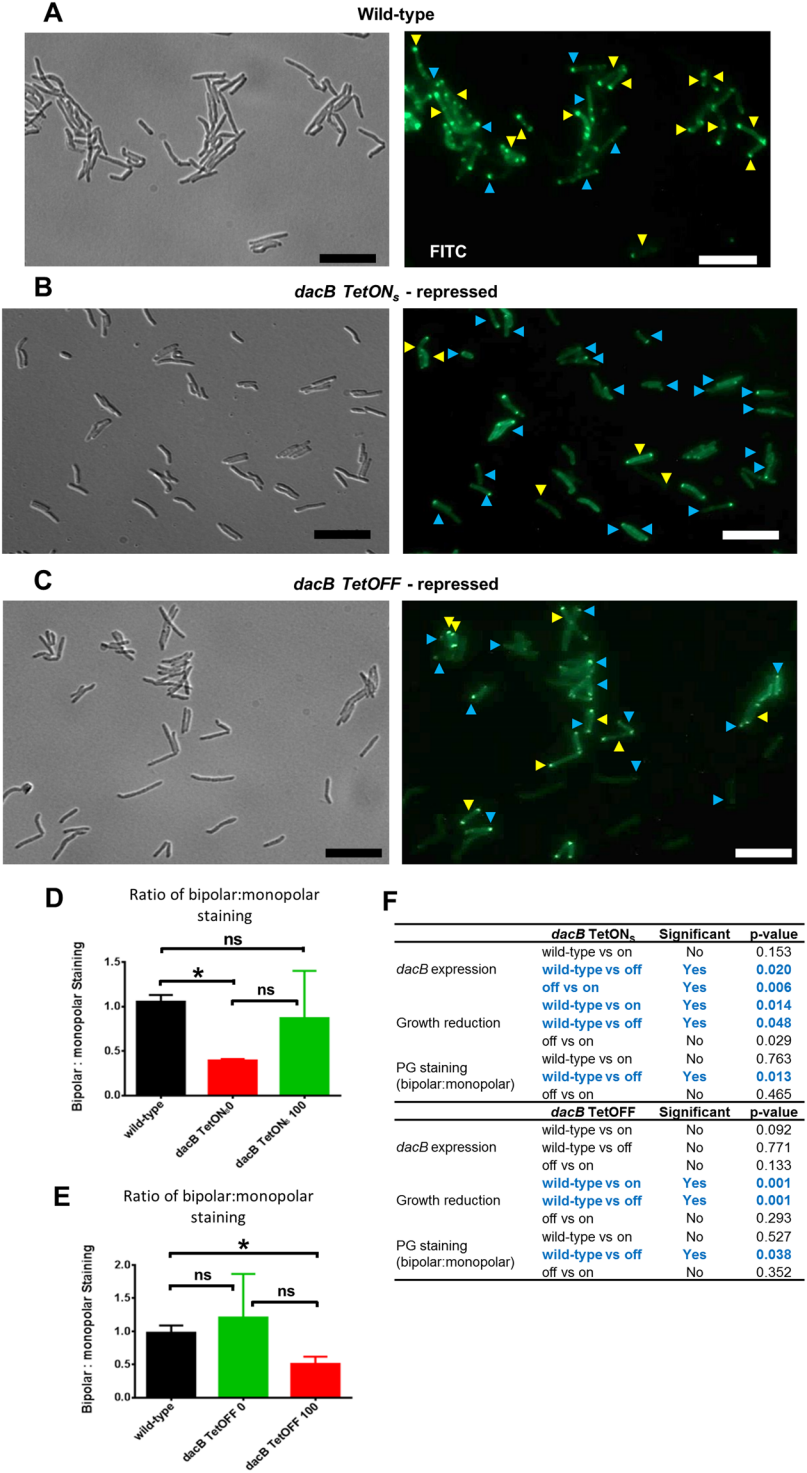
Repression of the *dacB* operon alters polar BODIPY-vancomycin staining. Strains were fluorescently labelled with BODIPY-vancomycin and scored for bipolar or monopolar staining. (**A**–**C**) Representative images for wild-type, *dacB* TetON_s_ 0 ng/ml aTc (repressed) and *dacB* TetOFF 100 ng/ml aTc (repressed) respectively. Yellow and blue arrow heads indicate bipolar and monopolar PG staining patterns, respectively. Scale bar = 10 µm. For additional fields of view of each strain, refer to Supplementary Fig. S9. In both strains, where the *dacB* operon was repressed, the ratio of number of cells with bipolar:monopolar staining was determined and plotted as a function of the inducer and compared to wild-type. These data are shown for *dacB* TetON_s_ in (**D**) and for *dacB* TetOFF in (**E**). In all cases, the wild-type strain served as a control and unpaired t-tests were used to detect statistically significant changes (*p < 0.05). (**F**) Table summarizing all statistical comparisons performed with associated p-values. Data are representative of three independent biological repeats in panels (**A**,**C**), with the exception of (**B**), which is representative of two.

During late logarithmic growth, the ‘forward’ repressor was able to significantly repress expression, with a statistically significant reduction in gene expression in the absence of aTc. Expression levels in the wild-type strain and *dacB* TetON_s_ 100 were not statistically significant (Fig. 4B). With the ‘reverse’ repressor, repression of *dacB* expression was evident but did not reach statistically significant levels (Fig. 4C). In both systems, a reduction in gene repression was associated with a reduced growth rate during mid- to late-logarithmic growth phases relative to the wild-type strain (Fig. 4D,E). However, these changes in growth rate for both the *dacB* TetON_s_ and *dacB* TetOFF strains were marginal and did not appear to be strongly dependent on the inducer. Hence, no definitive conclusions could be drawn. We next assessed the spatial distribution of new PG biosynthesis using a BODIPY-vancomycin conjugate and observed a predominant bi-polar staining pattern of BODIPY-vancomycin in the majority of wild-type cells (Fig. 5A). In both knockdown systems, repression of the *dacB* gene cluster appeared to be associated with a reduction in the frequency of cells with bipolar staining patterns and a concomitant increase in staining at only one pole (monopolar, Fig. 5B,C). We assessed this effect qualitatively by plotting the ratio of bipolar:monopolar stained cells and in both repression systems, repression of the *dacB* gene cluster resulted in a reduced ratio when compared to wild-type (Fig. 5D,E). These changes in staining were statistically significant (Fig. 5F). Additional fields of view for each strain can be found in Supplementary Fig. S9. The wild-type strain containing pMC1s or pTEK-4S0X alone was similarly stained with BODIPY-vancomycin during late-logarithmic growth. PG staining patterns (i.e. the predominance of bipolar over monopolar staining) remained unaltered when compared to the parental mc^2^155 strain (Supplementary Fig. S10).

## Discussion

The genetic multiplicity of LMW PBPs in a variety of bacterial organisms has presented a long standing conundrum in bacterial physiology that remains unresolved. Whilst the role of PBP5 (or similar homologues) in other organisms with respect to maintenance of cell morphology and modification of PG is established^7,8,10,12,14,37^, the mechanistic basis of how these functions relate to the acylserine transferase activity of DD-CPases is still unclear. In this study, we investigated the role of putative DD-CPase-encoding genes in mycobacterial growth. Bioinformatics analysis identified 4 putative DD-CPase encoding genes in *M. smegmatis*, all retaining the catalytic Ser-Xaa-Xaa-Lys (S*xx*K), Ser-Xaa-Asn (S*x*N) and Lys-Thr-Gly (KTG) motifs, which are required to coordinate the residues of the incoming stem peptide^5^.

Transcriptional analysis suggested DD-CPase-encoding genes were expressed to varying degrees during the growth phases tested. Relative expression of MSMEG_2433 and MSMEG_1661 transcripts were significantly higher, suggesting possible specialist roles. In *E. coli*, the most abundant LMW PBPs in the cell are DD-CPases. However, deletion of PBP4, PBP6 and DacD did not dramatically alter growth kinetics or cell morphology^7^, suggesting a disconnect between transcript abundance and essentiality for growth. In contrast, deletion of PBP5 in *E. coli* was associated with altered biofilm formation, presumably due to disturbances in overall surface topology, arrangement of surface proteins or defective packaging of flagella^38,39^. In our single deletion mutants, no growth/survival, cellular morphology and surface topology or biofilm defects were observed, corroborating earlier studies regarding mycobacterial DD-CPases^40,41^. In the study by Pandey *et al*.^40^, simultaneous deletion of two DD-CPases altered PG cross-linking through a shift in 3-3 to predominantly 4-3 cross-linking. An associated increase in β-lactam sensitivity was also observed suggesting that DD-CPases regulate the substrate availability of LD-transpeptidases. Baranowski *et al*.^42^ recently demonstrated that MSMEG_2433 (DacB2) possessed DD-carboxy- and endopeptidase activity. DacB2-mRFP localized to the side walls, which retain the older PG, together with LD-transpeptidases. In our study, loss of DacB2 did not yield defects in spatial localization of new PG units, suggesting that combinatorial deletion of DD-CPases with other PG remodeling enzymes may give useful insight. In this regard, deletion of these in combination (MSMEG_2432 and MSMEG_2433^40^) or together with other proteins (PknI and DacB2^41^), yielded cell wall and morphology related abnormalities. In addition, two of the three DD-CPase homologues (DacB2 and Rv3627c) in *M. tuberculosis* were shown to be regulated, via the cytoplasmic redox potential and intracellular redox sensor, WhiB4, in response to Augmentin suggesting important roles in β-lactam tolerance^43^. These observations suggest that DD-CPases likely exert divergent functions, through interaction with other proteins.

The predicted essentiality of the *M. tuberculosis* homologue, Rv3627c^44,45^, suggests a possible divergence of physiological function in mycobacteria as no other bacterial genome encodes essential LMW PBPs. The homologous gene in *M. smegmatis*, MSMEG_6113 (*dacB*) is part of an identical four-gene operon encoding MSMEG_6113 (*dacB*), MSMEG_6112, *hpt* and *tilS*. Three of the genes in the homologous *M. tuberculosis* operon (Rv3637c-Rv3626c-*mesJ*-*hpt*) are predicted to be essential while Rv3626c is dispensable^44,45^. We attempted to prove the essentiality of MSMEG_6113 (*dacB*) by deleting the native allele in the chromosome only in the presence of additional copy of the homologous *M. tuberculosis* operon. We were unable to generate a deletion mutant in the presence of an additional copy of the *dacB* gene alone and it is plausible that the gene contains regulatory elements required by downstream, essential genes. Repression of this operon using tetracycline repressors^36,46^, yielded a reduction in growth and reduced frequency of cells with bipolar incorporation of new PG units. Given that these effects may be associated with reduction of *dacB* alone, or with reduction of the other genes in this operon, and that the growth defects observed were marginal at best, further work is required to resolve this observation. In this regard, our attempts to generate in-frame *dacB* deletions, with a variety of complementing vectors which include different components of the operon, were unsuccessful. Only genetic complementation with the full operon allows for recovery of a mutant *dacB* allele at the native site. Hence, the genetic intractability of this system has made it difficult to dissect out these effects further.

Collectively, this study has demonstrated that three of the four putative DD-CPases in *M. smegmatis* are dispensable for growth, whilst the *dacB* operon is essential. Further work with combinatorial mutants may yield useful insight into the biology of this highly conserved group of proteins.

## Methods

### Bacterial strains, media and culture conditions

The bacterial strains used in this study are listed in Table S1. *E. coli* strains were grown in Luria-Bertani broth (LB) or on solid Luria agar (LA). Unless otherwise indicated, *M. smegmatis* strains were grown in Middlebrook 7H9 media (Difco) supplemented with 0.2% glycerol, glucose salts (1X) and 0.05% Tween 80. Kanamycin (Kan) and Hygromycin (Hyg) were used in *E. coli* cultures at a final concentration of 50 and 200 µg/ml, respectively and in *M. smegmatis* cultures at 25 and 50 µg/ml, respectively.

### Allelic exchange mutagenesis

The plasmids and primer pairs used in this study are detailed in Tables S1 and S2, respectively. Suicide plasmids to introduce in-frame, unmarked deletions were constructed according to the protocols outlined in Parish and Gordhan^47^. This approach yielded p2∆*dacB*, p2∆2432, p2∆2433 and p2∆1661 as knockout vectors for site-specific deletion of *dacB*, MSMEG_2432, MSMEG_2433 and MSMEG_1661 respectively. Attempts to delete *dacB* also required cloning of the *lacZ-sacB* cassette, excised from pGOAL17 by *Pac*I digestion, into the p2NIL vector, carrying the upstream and downstream homologous regions, to create p2∆*dacB*g17. Knockout vectors were pre-treated by UV irradiation^48^, electroporated into *M. smegmatis* mc^2^155 and allelic exchange mutants were recovered by two-step selection as previously described^47^. PCR screening for putative mutants was carried out using gene-specific primers (Table S4).

A *dacB* complementation vector, pMV*dacB*, was constructed by cloning the full-length *dacB* with 150 bp region upstream of the start codon into pMV306H, and the resulting vector was electroporated into *M. smegmatis* to create mc^2^155 *attB*::pMV*dacB*. A merodiploid strain was constructed by electroporating the single crossover (SCO) strain with the complementation plasmid pMV*dacB* followed by selection for kanamycin- and hygromycin-resistant transformants. Integration of the pMVdacB was confirmed using PCR with the *attB* primer pairs (Table S5) the resulting recombinant, post sucrose counter selection, were screened using PCR with gene-specific primers (Table S4). A second complementation vector, pMVRv3627c_operon was constructed by digesting a BAC (Bacterial Artificial Chromosome) library containing DNA fragments from *M. tuberculosis* with *MluI*. The resulting fragment was cloned into pMV306H, and the resulting vector was electroporated into the SCO strain to yield ∆*dacB attB*::pMVRv3627c_operon. Allelic exchange mutants were recovered by two-step selection as previously described^47^.

### Conditional expression of *dacB*

Three hundred and fifty nucleotides corresponding to the 5′ region of *dacB* together with a 15 bp upstream region presumed to contain the native ribosome binding site (rbs) was PCR amplified from *M. smegmatis* mc^2^155 genomic DNA using the primers described in Table S2. This fragment was cloned downstream of the *P*_*myc1*_*tetO* promoter-operator element^36^ to create pSE_*dacB*, which was electroporated into *M. smegmatis* to create an SCO strain. Conditional anhydrotetracycline (aTc) regulation required the introduction of the tetracycline repressor (*tetR*) into this strain. Two variants of the repressor were used in this study (i) the wild-type TetR, expressed from a strong promoter – termed TetON_s_ carried on pMC1s^36^ and (ii) the reverse TetR (r1.7), carried on pTEK-4S-0X expressed from a strong mycobacterial promoter^46^ – termed TetOFF. Positive transformants were confirmed with PCR and southern hybridization.

### Growth kinetics of *dacB* depletion strain

The *dacB* TetON_s_ depletion strain was pre-cultured in 7H9 media supplemented with kanamycin (25 µg/ml), hygromycin (50 µg/ml) and aTc (5 ng/ml – to prevent the emergence of early tetracycline escape mutants) and grown to late logarithmic or stationary phase. A pre-culture for *dacB* TetOFF was prepared similarly but without aTc. Where necessary, aTc was removed from the pre-culture by washing the bacterial cells three times with 7H9 media lacking antibiotics (4500 rpm for 10 min at 4 °C). Pellets were re-suspended in 5 ml 7H9 media and used to inoculate cultures with varying concentrations of aTc (0 or 100 ng/ml). The wild-type and repressor-only strains served as controls. Samples were removed at 6 (early logarithmic) and 18 hours (late-logarithmic) for analysis by scanning electron microscopy (SEM) and fluorescence microscopy.

### Microscopy

#### Scanning Electron Microscopy

Bacterial cells were harvested at pre-determined time-points and harvested by centrifugation, washed twice with PBS and re-suspended in 2.5% glutaraldehyde in 1X PBS overnight at 4 °C. The cells were then washed twice with PBS and re-suspended in 100 μl 2% osmium tetroxide in PBS and incubated at room temperature for 1 hour, followed by dehydration in a series of ethanol concentrations for 2 min at each concentration beginning with 30% then 50%, 70% and twice at 100% ethanol followed by storage in 100% ethanol. Cells were spotted on a filter, coated twice with carbon and viewed using the FEI Nova NanoSEM 230.

#### Fluorescence Microscopy

For visualization of fluorescent tags, bacteria were incubated with labeled BIODIPY-Vancomycin (Invitrogen) according to the manufacturer’s instructions. Unlabeled vancomycin was added to a final concentration of 0.25 µg/ml. Samples were incubated at 37 °C for 1.5 hours. Unincorporated vancomycin was removed by washing cells followed by re-suspension in 50 µl 7H9. Ten to fifteen microliters were spotted onto 2% agarose pads and samples were viewed using a 100 ×, 1.46 numerical aperture objective mounted to an Axio Observer Z1 base (Zeiss). Images were taken in bright-field (DIC) and FITC channels using an AxioCam HRm camera and processed using the Zen blue Ver 5.1.2600 (Zeiss). Images were manipulated for brightness and contrast using the ImageJ V 1.8 to allow for clear visualization of specific dyes. These manipulations were applied to the entire image. In some cases, representative bacteria are shown for quantification purposes.

### Quantitative real-time PCR

RNA was extracted at pre-determined time-points using the NucleoSpin® RNA II kit (Machery and Nagel) as per the manufacturer’s instructions. RNA conversion to cDNA was performed using 1 µg DNAse-treated (Ambion) RNA and SuperScript III (Invitrogen) as per the manufacturer’s instructions. Real-time qPCR analysis was performed using Evagreen Sofast Supermix (BioRad) as per the manufacturers’ instructions using the primer sets listed in Table S2. Absolute numbers of transcript were normalized to the number of *sigA* transcripts in the same sample and the normalized data were compared to the normalized data in the wild-type samples.

## Supplementary information

Supplementary information_SREP-15-19447A

## Data Availability

All data generated or analyzed during this study are included in this published article (and its Supplementary Information Files).
