# Supplementary material for: Characterization of putative DD-carboxypeptidase-encoding genes in Mycobacterium smegmatis
Source: Sci Rep. 2019 Mar 26;9:5194. doi: 10.1038/s41598-019-41001-x (PMC6435803; doi:10.1038/s41598-019-41001-x)
Supplement: Supplementary file 1 — Supplementary information_SREP-15-19447A [file 41598_2019_41001_MOESM1_ESM.pdf]

Characterization of putative DD-carboxypeptidase-encoding genes in *Mycobacterium smegmatis*

Christopher S. Ealand[1], Rukaya Asmal[1], Lethabo Mashigo[1], Lisa Campbell[1] and Bavesh D. Kana[1,2†]

SUPPLEMENTARY INFORMATION

[1]DST/NRF Centre of Excellence for Biomedical TB Research, School of Pathology, Faculty of Health Sciences, University of the Witwatersrand and the National Health Laboratory Service, P.O. Box 1038, Johannesburg, 2000, South Africa

[2]MRC-CAPRISA HIV-TB Pathogenesis and Treatment Research Unit, Centre for the AIDS Programme of Research in South Africa, CAPRISA, Durban, South Africa

Running title: Putative DD-carboxypeptidases in mycobacteria

†For correspondence: Mailing address: DST/NRF Centre of Excellence for Biomedical TB Research, National Health Laboratory Service, P. O. Box 1038, Johannesburg 2000, South Africa. Phone: Tel: + (27) 11 4899135; Fax: + (27) 11 4899397; E-mail: bavesh.kana@nhls.ac.za (B.D.K)

Supplementary Tables

Table S1. Bacterial strains and plasmids used in this study.

Name	Description	Source/Reference
Strains		
M. smegmatis		
mc^{2}155	*ept-1* (efficient plasmid transformation) mutant of mc^{2}6	[1]
Δ2432	Derivative of mc^{2}155 carrying an unmarked, out-of-frame deletion in *M. smegmatis* Msmeg_2432 (786bp internal region excised)	This work
Δ2433	Derivative of mc^{2}155 carrying an unmarked, out-of-frame deletion in *M. smegmatis* Msmeg_2433 (870bp internal region excised)	This work
Δ1661	Derivative of mc^{2}155 carrying an unmarked, in-frame deletion in *M. smegmatis* Msmeg_1661 (1158bp internal region excised)	This work
Δ*dacB* TetO	Derivative of mc^{2}155 in which the native promoter of Msmeg_6113 (*dacB*) is substituted with Pmyc1tetO. The native promoter regulates expression of a truncated (350 bp) 5' region of *dacB*; Hyg^r. This strain contains no Anhydrotetracycline-responsive repressor in the L5 attB site thus termed repressor-less.	This work
Δ*dacB* TetON$_s$	Derivative of mc^{2}155 in which the native promoter of Msmeg_6113 (*dacB*) is substituted with Pmyc1tetO. The native promoter regulates expression of a truncated (350 bp) 5' region of *dacB*; Hyg^r. This strain contains the "strong" Anhydrotetracycline-responsive repressor (tetR) in the L5 attB site derived from pMC1s.	This work
Δ*dacB* TetOFF	Derivative of mc^{2}155 in which the native promoter of Msmeg_6113 (*dacB*) is substituted with Pmyc1tetO. The native promoter regulates expression of a truncated (350 bp) 5' region of *dacB*; Hyg^r. This strain contains the "reverse" Anhydrotetracycline-responsive repressor (revtetR) in the L5 attB site derived from pTEK-4S0X.	This work
Δ*dacB attB*::pMV*dacB*	Derivative of mc^{2}155 carrying native *dacB*, Δ*dacB* and additional copy of native *dacB* in the L5 *attB* site. Strain was constructed by electroporation with pMV*dacB* and p2Δ*dacB*g17 vectors; Hyg^r, Km^r.	This work
Δ*dacB* *attB*::pMVRv3627c_operon	Derivative of mc^{2}155 carrying native *dacB*, Δ*dacB* and additional copy of homologous operon from *M. tuberculosis* in the L5 *attB* site. Strain was constructed by electroporation with pMVRv3627c_operon and p2Δ*dacB*g17 vectors; Hyg^r, Km^r.	This work

<u>Plasmids</u>		
p2NIL	*E. coli* cloning vector, Kan^r	[2]
pMV306H	*E. coli*-Mycobacterium integrating shuttle vector. Integrates in the L5 *attB* site in mycobacteria. Derivative of pMV306 ([3]) carrying a *hyg* gene; Hyg^r	H. Boshoff
pMV*dacB*	Derivative of pMV306H carrying full-length *dacB* (1443 bp) plus 150 bp upstream of start codon representative of native promoter sequence; Hyg^r	This work
pMVRv3627c_operon	Derivative of pMV306H carrying four gene operon (Rv3627c, Rv3626c, Rv3625c (mesJ) and Rv3624c (hpt)) from *M. tuberculosis*; Hyg^r	This work
pMV1661	Derivative of pMV306H carrying full-length MSEMG_1661 (1194 bp) plus 150 bp upstream of start codon representative of native promoter sequence; Hyg^r	This work
pMV2432	Derivative of pMV306H carrying full-length MSMEG_2432 and MSMEG_2433 (1788 bp) plus 150 bp upstream of start codon representative of native promoter sequence; Hyg^r	This work
pMV2433	Derivative of pMV306H carrying full-length MSMEG_2433 (891 bp) plus 150 bp upstream of start codon representative of native promoter sequence; Hyg^r	This work
	Plasmid carrying *lacZ* and *sacB* markers as a *PacI* cassette; Km^r	[2]
pGOAL17	Plasmid carrying *lacZ-sacB-Hyg^r* markers as a *PacI* cassette; Hyg^r	[2]
pGOAL19<br>p2Δ2432	Knockout vector for creating ΔMsmeg_2432 mutant. The 1.2 kb upstream region including 18 bp of the 5' sequence of Msmeg_2432 was PCR-amplified to incorporate *HindIII* and *NdeI* restriction sites. The 1.2 kb downstream was similarly obtained but incorporated *NdeI* and *Acc65I r*estriction sites. The fragments were cloned into p2NIL and the *PacI* cassette from pGOAL19 was inserted; Km^r Hyg^r.	This work
p2Δ2433	Knockout vector for creating ΔMsmeg_2433 mutant. The 1.2 kb upstream region including 18 bp of the 5' sequence of Msmeg_2433 was PCR-amplified to incorporate *HindIII* and *NdeI* restriction sites. The 1.2 kb downstream was similarly obtained but incorporated *NdeI* and *Acc65I r*estriction sites. The fragments were cloned into p2NIL and the *PacI* cassette from pGOAL19 was inserted; Km^r Hyg^r.	This work
p2Δ1661	Knockout vector for creating ΔMsmeg_1661 mutant. The 1.2 kb upstream region including 18 bp of the 5' sequence of Msmeg_2433 was PCR-amplified to incorporate *HindIII* and *NdeI* restriction sites. The 1.2 kb downstream was similarly obtained but incorporated *NdeI* and *Acc65I r*estriction sites. The fragments were cloned into p2NIL and the *PacI* cassette from pGOAL19 was inserted; Km^r Hyg^r.	This work
p2Δ*dacB*	Knockout vector for creating ΔMsmeg_6113 (*dacB*) mutant. The 1.2 kb upstream region including 18 bp of the 5' sequence of Msmeg_2433 was PCR-amplified to incorporate *HindIII* and *NotI* restriction sites. The 1.2 kb downstream was similarly obtained but incorporated *NotI* and *Acc65I r*estriction sites. The	This work

	fragments were cloned into p2NIL and the *PacI* cassette from pGOAL19 was inserted; Km^r Hyg^r.	
p2Δ*dacB*g17	Knockout vector for creating ΔMsmeg_6113 (*dacB*) mutant. The 1.2 kb upstream region including 18 bp of the 5' sequence of Msmeg_2433 was PCR-amplified to incorporate *HindIII* and *NotI* restriction sites. The 1.2 kb downstream was similarly obtained but incorporated *NotI* and *Acc65I r*estriction sites. The fragments were cloned into p2NIL and the *PacI* cassette from pGOAL17 was inserted; Km^r.	This work
pSE100	Derivative of pMS2 containing Pmyc1tetO carrying a *hyg* gene; Hyg^r. pMS2 is a shuttle vector containing origins of replication for *Escherichia coli* (pMB1) and mycobacteria (pAL500) and a multiple cloning site flanked by two transcriptional terminators.	Sabine Erht
pMC1s	Derivative of pMV306K, which is a single-copy-integrating plasmid which inserts into L5 attB; Km^r. Anhydrotetracycline-responsive repressor (tetR) is not codon-usage adapted for mycobacteria but is expressed from a strong mycobacterial promoter (psmyc = puv15).	Sabine Ehrt
pTEK-4S0X	Kanamycin resistant derivative of pTE-4S0X; Km^r. Anhydrotetracycline-responsive repressor (revtetR) is expressed from a strong mycobacterial promoter (psmyc = puv15). High-copy number in *E. coli* and low-copy number in mycobacteria.	Sabine Ehrt
pS*dacB*	Derivative of pSE100. Suicide construct from which the mycobacterial ori was excised and the native promoter (15 bp upstream start codon) and 350 bp of the 5' region of *dacB* cloned directly downstream of the Pmyc1tetO using *SphI* and *MscI*; Hyg^r.	This work

Table S2. Primers used to generate suicide plasmids for MSMEG_6113 (*dacB*), MSMEG_2432, MSMEG_2433 and MSMEG_1661.

**Region amplified**	**Primer name**	**Sequence (5' – 3')[a]**	**Description of amplicon**	**Mutation**
Msmeg_6113 (*dacB*) upstream region	dacB_UF	5' – CGCGAAGCTT GCGAGCCCCTGGTGAACC	1200 bp of upstream and 21 bp of the 5'-end of Msmeg_6113 (*dacB*)	1400 bp unmarked deletion in *dacB*
	dacB_UR	5' – CGCGGCGGCCGCGCGCGTCAGCCTATCGCTCAT		
Msmeg_6113 (*dacB*) downstream region	dacB_DF	5' – CGCGGCGGCCGCCAGCTGCGGATGCGGCGCATGA	1339 bp of downstream and 22 bp of the 3'-end of Msmeg_6113 (*dacB*)	
	dacB_DR	5' – CGCGGGTACCATCTCGGCGCCAACACAT		
Msmeg_1661 upstream region	1661_UF	5' – CGCGAAGCTTGCGAGGCCGTCGAGAGCG	1200 bp of upstream and 18 bp of the 5'-end of Msmeg_1661	1158 bp unmarked deletion in Msmeg_1661
	1661_UR	5' – CGCGCATATGCACCGCGCGTCGCGTCAT		
Msmeg_1661 downstream region	1661_DF	5' – CGCGCATATGCAGCGCCCGCAGCACTGA	1375 bp of downstream and 18 bp of the 3'-end of Msmeg_1661	
	1661_DR	5' – CGCGGGTACCATTCGGGTTGTCGCGGTT		
Msmeg_2432 upstream region	2432_UF	5' – CGCGAAGCTTCCGGGAACGCAGGCAGC	1200 bp of upstream and 21 bp of the 5'-end of Msmeg_2432	786 bp unmarked deletion in Msmeg_2432
	2432_UR	5' – CGCGCATATGCGCCGCGAACAGTCTTCGCAC		
Msmeg_2432 downstream region	2432_DF	5' – CGCGCATATGGACTACGGCTTCAGCCAGTAG	1200 bp of downstream and 18 bp of the 3'-end of Msmeg_2432	
	2432_DR	5' – CGCGGGTACCGGCCGACTTCGACCTGTCG		
Msmeg_2433 upstream region	2433_UF	5' – CGCGAAGCTTACGATCCCGCGGACCAGG	1200 bp of upstream and 21 bp of the 5'-end of Msmeg_2433	870 bp unmarked deletion in Msmeg_2433
	2433_UR	5' – CGCGCATATGCCCGAAGGCGTACCTCCACAT		
Msmeg_2433 downstream region	2433_DF	5' – CGCGCATATGGCGAGCATCGGGGCGCTCTGA	1200 bp of downstream and 21 bp of the 3'-end of Msmeg_2433	
	2433_DR	5' – CGCGGGTACCATCGGCCACGGCCGCATC		

a. Restriction sites are underlined.

Table S3. Primers used for gene expression analysis by qPCR.

**Gene**	**Primer name**	**Sequence (5’ – 3’)**	**Amplicon size (bp)**
Msmeg_6113 (*dacB*)	*dacB*_qF	5’ – AACGAGTCGGACAACGTGAT	Positions 934-1140 (207 bp)
	dacB_qR	5’ – GACCACCTCATCGAGGGTAA	
Msmeg_1661	1661_qF	5’ – CCACACCCAGAGCTACCAGT	Positions 732-924 (193 bp)
	1661_qR	5’ – GGTTCCCTTGAGCAACACC	
Msmeg_1900	1900_qF	5’ – GACAACGCGGTTCAGACCTA	Positions 313-491 (179 bp)
	1900_qR	5’ – CAGGATTCGTTGGCGTAGAT	
Msmeg_2432	2432_qF	5’ – GCTGAACTATCCGGTGATCG	Positions 567-768 (202 bp)
	2432_qR	5’ – GTACATCTGCACCACCACCA	
Msmeg_2433	2433_qF	5’ – GCTGGTCAACCAGAACGAGT	Positions 651-803 (153 bp)
	2433_qR	5’ – TCTTTCACCAGGCCGTACAT	

Table S4. Primers used for genotyping *M. smegmatis* mutant strains. Primers F and R2 situated 100 bp upstream and downstream of start and stop codons, respectively. Primer R1 approximately in the middle of gene targeted for deletion.

**Gene**	**Primer name**	**Sequence (5’ – 3’)**	**Amplicon sizes (bp)**
Msmeg_6113 (*dacB*)	KO*dacB*_F	5’ – ACGACGTCGAACTCCACC	FR1 = 800 bp (wild-type)
	KO*dacB*_R1	5’ – CGTCGATGTCGGCCGGGTC	FR1 = absent (Δ)
	KO*dacB*_R2	5’ – ATGGCCTGGCGGCGCGTG	FR2 = 1643 bp (wild-type)
			FR2 = 242 bp (Δ)
Msmeg_1661	KO1661_F	5’ – CACACGAGTCACAACTGT	FR1 = 701 bp (wild-type)
	KO1661_R1	5’ – TGGCGGCCAGCGTGTTGA	FR1 = absent (Δ)
	KO1661_R2	5’ – CGGCATGGTGAACCAGTC	FR2 = 1394 bp (wild-type)
			FR2 = 236 bp (Δ)
Msmeg_2432	KO2432_F	5’ – GGACCGTTCGGCGAGCAT	FR1 = 500 bp (wild-type)
	KO2432_R1	5’ – TGTCGGCGAGCATGTTGG	FR1 = absent (Δ)
	KO2432_R2	5’ – CGGATCTGGTGATCCTGC	FR2 = 1028 bp (wild-type)
			FR2 = 242 bp (Δ)
Msmeg_2433	KO2433_F	5’ – GCAGTCGAATAGTCCACC	FR1 = 500 bp (wild-type)
	KO2433_R1	5’ – CCGCGTCGTTGCCCGACG	FR1 = absent (Δ)
	KO2433_R2	5’ – AGTGCCGTGCTGAGGGCG	FR2 = 1028 bp (wild-type)
			FR2 = 242 bp (Δ)

Table S5. Primers used to amplify Msmeg_6113 (*dacB*) for complementation and for construction and confirmation of merodiploid strain in *M. smegmatis*.

Gene	Primer name	Sequence (5' – 3')[a]	Description	Amplicon size (bp)
MSMEG_6113 (*dacB*)	Comp_*dacB*_F<br>Comp_*dacB*_R	5'–GCGCACGCGTTCTCGTGATCCACCTCGT–3'<br>5'–GCGCCTGCAGTCATGCGCCGCATCCGCA–3'	150 bp upstream of 5'–end amplified with full-length *dacB* to maintain native promoter sequences (-10 and -35)	1593 bp
MSMEG_1661	Comp_1661_F<br>Comp_1661_R	5'–GCGCACGCGTGAGAAGTTCGAGTAACGC–3'<br>5'–GCGCACGCGTTCAGTGCTGCGGGCGCTG–3'	150 bp upstream of 5'–end amplified with full-length MSMEG_1661to maintain native promoter sequences (-10 and -35)	1364 bp
MSMEG_2432	Comp_2432_F<br>Comp_2432_R	5'–GCGCAAGCTTCGTCCGGGGTGAACACCG–3'<br>5'–GCGCAAGCTTCTACTGGCTGAAGCCGTA–3'	150 bp upstream of 5'–end amplified with full-length MSMEG_2433 and MSMEG_2432 to maintain native promoter sequences (-10 and -35)	1958 bp
MSMEG_2433	Comp_2433_F<br>Comp_2433_R	5'–GCGCAAGCTTCGTCCGGGGTGAACACCG–3'<br>5'–GCGCAAGCTTTCAGAGCGCCCCGATGCT–3'	150 bp upstream of 5'–end amplified with full-length MSMEG_2433to maintain native promoter sequences (-10 and -35)	1061 bp
attB (tRNA for glycine)	attL2_F<br>attBS1_R	5' – CTT GGA TCC TCC CGC TGC GC – 3'<br>5' – ACG TGG CGG TCC CTA CCG – 3'	Site for integration of the complementation vector pMV*dacB* in the bacterial chromosome	282 bp
	attBS2_F<br>attL4_R	5' – ACA GGA TTT GAA CCT GCG GC – 3'<br>5' – AAT TCT TGC AGA CCC CTG GA – 3'		320 bp

a. Restriction sites are underlined.

Table S6. Primers used to generate suicide plasmid for use in constructing Msmeg_6113 (*dacB*) promoter replacement strain in *M. smegmatis*.

Gene	Primer name	Sequence (5' – 3')[a]	Description	Amplicon size (bp)
Msmeg_6113 (*dacB*)	KD_*dacB*_F	5' – GCGCATGCCCGGGCCAACCTTAGATGAGCGATA	15 bp upstream of start codon and 350 bp of 5'–end harbouring putative native promoter (-10) sequence	393 bp
	KD_*dacB*_R	5' – GCGCCCCGGGCCCGTGTCGGCGTCGGTG		

a. Restriction sites are underlined and start codons are indicated in bold.

Supplementary Figures

```
Mtb_dacB1            -------------------------MAFLRSVSCLAAAVFAVGTGIGLPTAAGEPNAAPA
Mtb_dacB2            ------------------------------------------------------------
Mtb_Rv3627c          --------------------MGPTRWR-KSTHVVVGAAVLAFVAVVVAAAALVTTG----
MSMEG_dacB           MSDRLTRRGGSPRSIESRGSMRPTRWR-RSTHVAVGVAVLALVVAVVAAAALF-TG----
MSMEG_2432           ------------------------------------------------------------
MSMEG_2433           ------------------------------------------------------------
MSMEG_1661           -------------------------MT-RRAVTAVGALALLTAPLMTAGVAGAEPG---V
MSMEG_1900           ------------------------------------------------------------
E.coli_dacA          ------------------------------------------------------------
E.coli_dacB          ------------------------------------------------------------
E.coli_dacC_PBP6     ------------------------------------------------------------
E.coli_dacD_PBP6b    ------------------------------------------------------------

Mtb_dacB1            ACPYKVSTP--PAVDSSEVPAAGE-P-PLPLVVPPTPVGGNALGGCGIITA--PGSAPAP
Mtb_dacB2            MRKLMTATA--ALCACAVTVSAGA------------AWADADVQPAGSVPI--PDGP---
Mtb_Rv3627c          GHRAGVRAP--APPPRPPTVKAGV---------VPVADTAATPSAAGVTAA--LAVVAAD
MSMEG_dacB           KHSDAAEAV--PPAPPPATADPGV---------VPVDLSAPTPTRRGLATA--LAAALAN
MSMEG_2432           MRRLFAAAA--FALSTALAAATFT----------PVAHAEPAAAPAGAAV--TDGP---
MSMEG_2433           MWRYAFGLV--VLVVSGLITGPGSLA-------VPVARADADIQQVGSVAP--PEGP---
MSMEG_1661           DCPYRETTP--PAVDASEVPKPGE-AAPGPLPVPPKAIGGEALSGCGVITA--PNTPPVP
MSMEG_1900           ------------------------------------------------------------
E.coli_dacA          -----MNTIFSARIMKRLALTTAL----------CTAFISAAHADDLNIKTMIPGVP---
E.coli_dacB          -----------MRFSRFIIGLTSC-----------IAFSVQAANVDEYITQ-LPAGA---
E.coli_dacC_PBP6     -----MTQY--SSLLRGLAAGSAF----------LFLFAPTAFAAEQTVEA-----P---
E.coli_dacD_PBP6b    -------------MKRRLIIAASL----------FVFNLSSGFAAENIPFS--PQPP---

Mtb_dacB1            GDVSAEAWLVADLDSGAVIAARDPHGRHRPASVIKVLVAMASINTLTLNKSV--------
Mtb_dacB2            ----AQTWIVADLDSGQVLAGRDQNVAHPPASTIKVLLALVALDELDLNSTV--------
Mtb_Rv3627c          PDLGKLAGRITDALTGQELWQRLDDVPLVPASTNKILTAAAALLTLDRQARISTRVVAGG
MSMEG_dacB           PDLGLITGRITDADTGAELWEQGARVPMQPASVNKVLTTAAALLTLDRDARLTTTVVAAD
MSMEG_2432           ----AKAWLVADMDTGRVLASKDPYGSYAPASTIKPLLAMVVLDHLRPDNFA--------
MSMEG_2433           ----AETWVVADMDTGQILAGRGEYVRHAPASTIKTLLALVVLDEVPLDSTI--------
MSMEG_1661           EDVSAEAWIVADMDTGDVIAARDPHGRHRPASIIKVLTATAALNELNLNKRV--------
MSMEG_1900           ------------------------------------------------------------
E.coli_dacA          -QIDAESYILIDYNSGKVLAEQNADVRRDPASLTKMMTSYVIGQAMKAGKFK--------
E.coli_dacB          ----NLALMVQKVGASAPAIDYHSQQMALPASTQKVITALAALIQLGPDFRF-TTTLETK
E.coli_dacC_PBP6     -SVDARAWILMDYASGKVLAEGNADEKLDPASLTKIMTSYVVGQALKADKIK--------
E.coli_dacD_PBP6b    -EIHAGSWVLMDYTTGQILTAGNEHQQRNPASLTKLMTGYVVDRAIDSHRIT--------

Mtb_dacB1            ------------------------------AGTADDAAVEGTKVGVNT--G---GTYTVN
Mtb_dacB2            ------------------------------VADVADTQAECNCVGVKP--G---RSYTAR
Mtb_Rv3627c          QNPQGPVVLVGAGDPTLSAAPPGQDTWYHGAARIGDLVEQIRRSGVTP--T---AVQVDA
MSMEG_dacB           DQP-GLVVLRGGGDTTLSAAPKGTDTWYKGAARISDLADQVRARGIRV--T---RVRVDT
MSMEG_2432           ------------------------------RANASHTKVECSCVGLKP--G---QPYTTR
MSMEG_2433           ------------------------------VADETDTDVECNCAGVAP--G---RTYTAR
MSMEG_1661           ------------------------------AGTQDDANAEGTRVGVGP--G---GQYTIN
MSMEG_1900           -------------------------------------------MGLGA--GLFGGLVTAS
E.coli_dacA          ---------------ETDLVTIGNDAW----ATGNPVFKGSSLMFLKP--G---MQVPVS
E.coli_dacB          GNV-ENGVLKGDL-----VARFGADPTLK-RQDIRNMVATLKKSGVNQIDG---NVLIDT
E.coli_dacC_PBP6     ---------------LTDMVTVGKDAW----ATGNPALRGSSVMFLKP--G---DQVSVA
E.coli_dacD_PBP6b    ---------------PDDIVTVGRDAW----AKDNPVFVGSSLMFLKE--G---DRVSVR

Mtb_dacB1            QLLHGLLMHSG--------NDAAYALARQLG--GMPAALEKINLLAAKLGGRDT--RVAT
Mtb_dacB2            QLLDGLLLVSG--------NDAANTLAHMLG--GQDVTVAKMNAKAATLGATST--HATT
Mtb_Rv3627c          SAFSGPTMAPGWDPADIDNGDIAPIEAAMID--AGRIQPTTVNSRRSRTPALDAGRELAK
MSMEG_dacB           SAYSGPTMAPGWDPADIDGGDIAPMESVMLD--GGRTQPTTVESRRSKSPALDAGKALAA
MSMEG_2432           QLLDALLMVSG--------NDAANMLADMLG--GPRVAVAAMNRKAAAVGARNT--RAAS
MSMEG_2433           QLLEAALLASG--------NDAANTLARMLG--GPEAAVAKMNAKAAQLGARDT--NVVT
MSMEG_1661           DLLHGLLMYSG--------NDAAHALAMQLG--GWDPALQKLNTLAAKLGGRDT--RAAT
MSMEG_1900           AVLTGALTAPA-PSPQIALVDNTEALTSSDGSLADGQAFSPFDLQQPAIAKLDP--RLLA
E.coli_dacA          QLIRGINLQSG--------NDACVAMADFAAG-SQDAFVGLMNSYVNALGLKNT--HFQT
E.coli_dacB          SIFASHDKAPGWPW-----NDMTQCF-------SAPPAAAIVDRNCFSVSLYSA------
E.coli_dacC_PBP6     DLNKGVIIQSG--------NDACIALADYVAG-SQESFIGLMNGYAKKLGLTNT--TFQT
E.coli_dacD_PBP6b    DLSRGLIVDSG--------NDACVALADYIAG-GQRQFVEMMNNYAEKLHLKDT--HFET
```

```
Mtb_dacB1           PSGLDGPGMS--TSAYDIGLFYRYAWQN-PV---FADIVATRTFDFPGHGDHPGYELEND
Mtb_dacB2           PSGLDGPGGSGASTAHDLVVIFRAAMAN-PV---FAQITAEPSAMFPS--DNGEQLIVNQ
Mtb_Rv3627c         ALGLDPAAVTI-ASAPAGARQLAVVQSA-PLIQRLSQMMNASDNVMA---ECIGREVAVA
MSMEG_dacB          ALGVEPESVTLMPSGMRGGTTIAEVQSA-PLIERLRQMMNESDNVMA---ESIAREVAEA
MSMEG_2432          PSGLDGPGWESLTTPHDLAVIFRAALNY-PV---IAQILRQTTAQFPG--KT----LTYQ
MSMEG_2433          PSGLDAPGMPFWSTPHDLAVIFRAAMAD-PV---FAQITAMPSTVFPA--KTGDRVLVNQ
MSMEG_1661          PSGLDGPGMS--TSAYDIALFYRYAWQN-PV---FADIVHTQSYQFPGR-DGGSYPIEND
MSMEG_1900          AVQNAANA----AAAEGITMTVTSGWRS-PA---FQQTLLDNAVQTYGS-LAAAREYVQT
E.coli_dacA         VHGLDADGQY--SSARDMALIGQALIRDVPN---EYSIYKEKEFTFNG------IRQLNR
E.coli_dacB         ------------PKPGDMAFIRVASYY--PV-TMFSQVRTLPRGSAEA--QYCELDVVPG
E.coli_dacC_PBP6    VHGLDAPGQF--STARDMALLGKALIHDVPE---EYAIHKEKEFTFNK------IRQPNR
E.coli_dacD_PBP6b   VHGLDAPGQH--SSAYDLAVLSRAIIHGEPE---FYHMYSEKSLTWNG------ITQQNR

Mtb_dacB1           NQLLYNYPG---ALGG-----KTGYTDDAGQTFVGAANRD-GRRLMTVLLHGTRQPIPP-
Mtb_dacB2           DELLQRYPG---AIGG-----KTGYTNAARKTFVGAAARG-GRRLVIAMMYGLVKEGGPT
Mtb_Rv3627c         INRPQSFSG---AVDAVTSRLNTAHIDTAGAALVDSSGLSLDNRLTARTLDATMQAAAGP
MSMEG_dacB          LGRPQSFEG---AVGAVLTQLRSVGIDTSGAKLVDSSGLSVDNRLTALTLDEVVNAAAGH
MSMEG_2432          NELLTRYPG---DIGG-----KTGYTNLARKTYVGAAQRG-NRRLVVVQMYG-----TGD
MSMEG_2433          NELLHRYPG---TLGG-----KTGFTDIARKTFVAAAQRD-GRRLVIAMMYGLVKEGGPT
MSMEG_1661          NKLLANYPG---ALGG-----KTGYTDDAGQTFVGAAERD-GRRLIAVLLKGTRVPIAP-
MSMEG_1900          PTASRHVTGEAVDIGG---------TGADQWLITN-----GPRFGLCQIYANE------
E.coli_dacA         NGLLWDNSL---NVDG----IKTGHTDKAGYNLVASATEG-QMRLISAVMGGRTFKGR--
E.coli_dacB         DLNRFTLTG---CLPQRSEPLPLAFAVQDGASYAGAILKD-ELKQAGITWSGTLLRQTQ-
E.coli_dacC_PBP6    NRLLWSSNL---NVDG----MKTGTTAGAGYNLVASATQG-DMRLISVVLGAKTDRIR--
E.coli_dacD_PBP6b   NGLLWDKTM---NVDG----LKTGHTSGAGFNLIASAVDG-QRRLIAVVMGADSAKGR--

Mtb_dacB1           -WEQAAHLLDYGFNTPAGTQIGTLIEPDPSLMSTDRNPADRQRVDP-QAAARISAADALP
Mtb_dacB2           YWDQAATLFDWGFALNPQASVGSL------------------------------------
Mtb_Rv3627c         DQPALRPLLDL---LPIAGGSGTL---GERFLDAATDQGPAGWLRA--KTGSLTAINSL-
MSMEG_dacB          TQPALRPLVDL---LPIAGGSGTL---SNRYLDTDAGRAAAGWLRA--KTGSLTGTNAL-
MSMEG_2432          LYDQAIRLFDYGFSQ---------------------------------------------
MSMEG_2433          YWDQAAGLLDWGFAQDRSASIGAL------------------------------------
MSMEG_1661          -WEQAARLLDYGFATPPGTKVGTLVDPDPSL-----APKQAEEPSPAQAASVLPPADAMP
MSMEG_1900          ---------SWHFEL-VADPLGNC----PPLLPDAAG-----------------------
E.coli_dacA         -EAESKKLLTWGFRF-----FETV----NPLKVGKEFASEPVWFGD-SDRASLGVDKDV-
E.coli_dacB         -VNEPGTVVASKQSAPLHDLLKIM------LKKSDNMIADTVFRMI--GHARFNVPGTW-
E.coli_dacC_PBP6    -FNESEKLLTWGFRF-----FETV----TPIKPDATFVTQRVWFGD-KSEVNLGAGEAG-
E.coli_dacD_PBP6b   -EEEARKLLRWGQQN-----FTTV----QILHRGKKVGTERIWYGD-KENIDLGTEQEF-

Mtb_dacB1           VRVGVAVIGALIVFGLIMVARAMNRRPQH-------------------------------
Mtb_dacB2           ------------------------------------------------------------
Mtb_Rv3627c         VGVLTDRSGRVLTFAFISNEAGPNGRNAMDALATKLWFCGCTT-----------------
MSMEG_dacB          AGIVTDRSGRVLTFALISNNAGPTGRTAIDALAAVLRSCGCGA-----------------
MSMEG_2432          ------------------------------------------------------------
MSMEG_2433          ------------------------------------------------------------
MSMEG_1661          VRVGVAVVGAIIVFMLILGARQLNQRPQH-------------------------------
MSMEG_1900          ------------------------------------------------------------
E.coli_dacA         -------------YLTIPRGRMKDLKASY-------------VLNSSELHAPLQKNQVVG
E.coli_dacB         ------RAGSDAVRQILRQQAGVDIGNTI-----IADGSGL---SRHNLIAPATMMQVLQ
E.coli_dacC_PBP6    -------------SVTIPRGQLKNLKASY-------------TLTEPQLTAPLKKGQVVG
E.coli_dacD_PBP6b   -------------WMVLPKAEIPHIKAKY-------------TLDGKELTAPISAHQRVG

Mtb_dacB1           ------------------------------------------------------------
Mtb_dacB2           ------------------------------------------------------------
Mtb_Rv3627c         ------------------------------------------------------------
MSMEG_dacB          ------------------------------------------------------------
MSMEG_2432          ------------------------------------------------------------
MSMEG_2433          ------------------------------------------------------------
MSMEG_1661          ------------------------------------------------------------
MSMEG_1900          ------------------------------------------------------------
E.coli_dacA         TINFQLDGKTIEQRPLVVLQEIPEGNFFGKIIDYIKLMFHHWFG----------------
E.coli_dacB         YIAQHDNE-------LNFISMLPLAGYDGSL-QY-RAGLHQ------AGVDGKVSAKTGS
E.coli_dacC_PBP6    TIDFQLNGKSIEQRPLIVMENVEEGGFFGRVWDFVMMKFHQWFGSWFS------------
E.coli_dacD_PBP6b   EIELYDRDKQVAHWPLVTLESVGEGSMFSRLSDY----FHHKA-----------------

Mtb_dacB1           ---------------------------------------------------------
Mtb_dacB2           ---------------------------------------------------------
```

```
Mtb_Rv3627c          ---------------------------------------------------------
MSMEG_dacB           ---------------------------------------------------------
MSMEG_2432           ---------------------------------------------------------
MSMEG_2433           ---------------------------------------------------------
MSMEG_1661           ---------------------------------------------------------
MSMEG_1900           ---------------------------------------------------------
E.coli_dacA          ---------------------------------------------------------
E.coli_dacB          LQGVYNLAGFITTASGQRMAFVQYLSGYAVEPADQRNRRIPLVRFESRLYKDIYQNN
E.coli_dacC_PBP6     ---------------------------------------------------------
E.coli_dacD_PBP6b    ---------------------------------------------------------
```

Figure S1A. Multiple sequence alignments for DD-CPase homologues. Shown are the DD-CPase homologues from *E. coli*, *M. tuberculosis* (Mtb) and *M. smegmatis* (MSMEG). The alignment was generated using Kalign (http://www.ebi.ac.uk/Tools/msa/kalign/). Conserved domains are highlighted in yellow (100% homologous) and turquoise (>= 75% homologous). The conserved Ser-Xaa-Xaa-Lys (S*xx*K), Ser-Xaa-Asn (S*x*N) and Lys-Thr-Gly (KTG) domains are shown in red boxes.

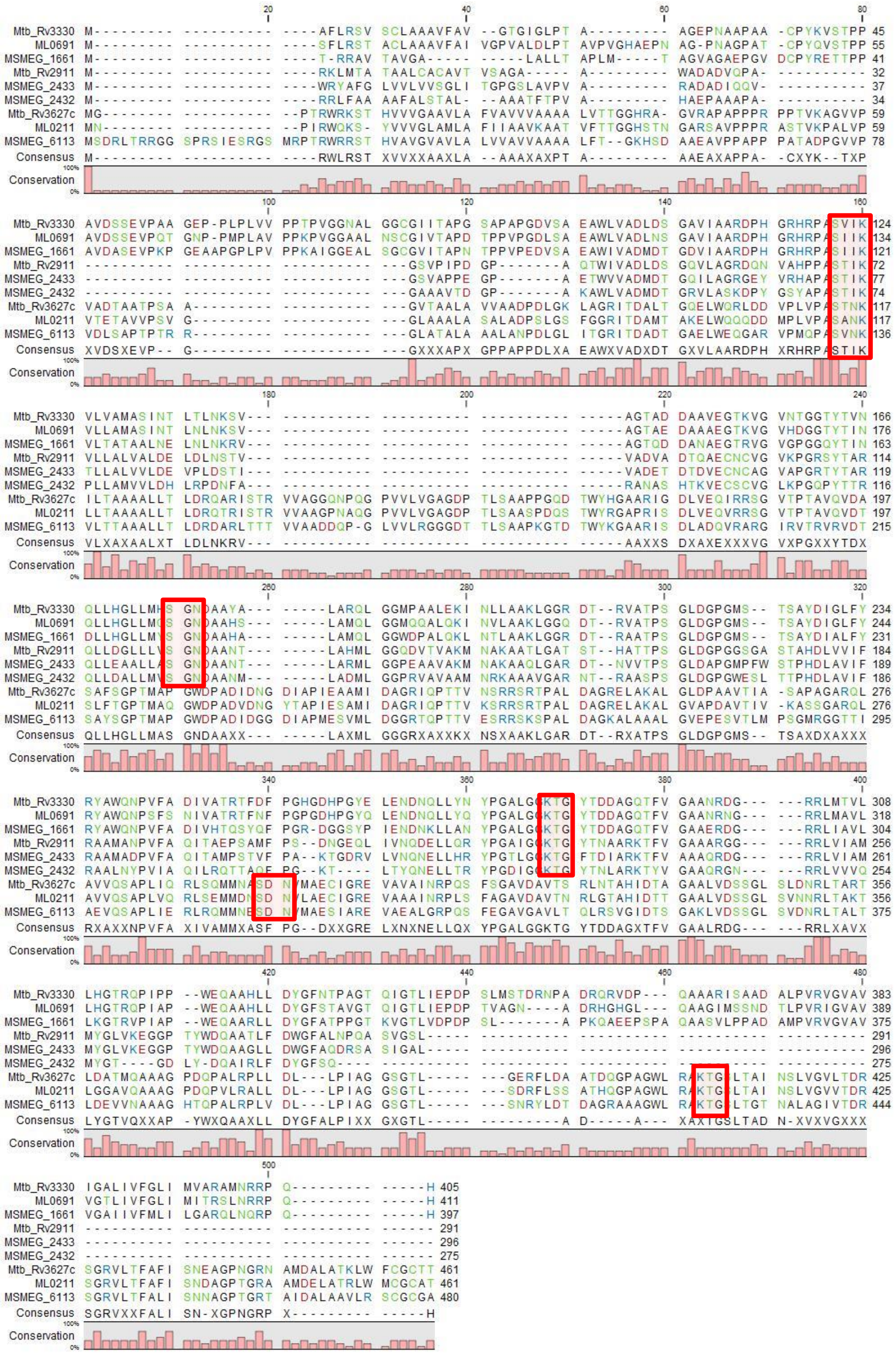

Figure S1B. Multiple sequence alignments for mycobacterial DD-CPase homologues. Shown are the DD-CPase homologues from *M. tuberculosis* (Mtb), *M. smegmatis* (MSMEG) and *M. leprae* (ML). The alignment was generated using CLC sequence viewer (Version 7.8). Conserved domains are highlighted in red boxes and correspond to the S*xx*K, S*x*N and KTG motifs required for DD-CPase activity.

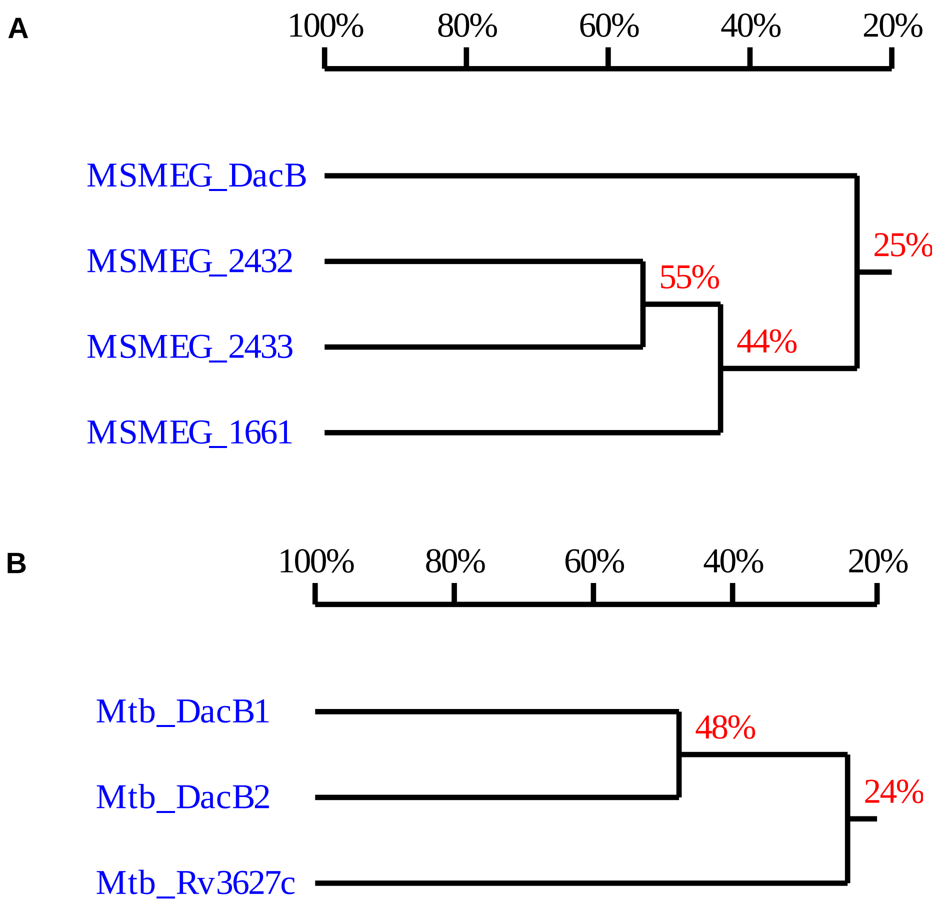

Figure S2. Homology trees for mycobacterial DD-CPase homologues. (A) Homology between four *M. smegmatis* mc2155 homologues. (B) Homology between three *M. tuberculosis* H37Rv homologues. In both cases, one homologue is located on a distinct branch in the phylogenetic tree (MSMEG_DacB and Rv3627c). Trees were compiled using DNAMAN software (version 4.13; Lynnon Biosoft).

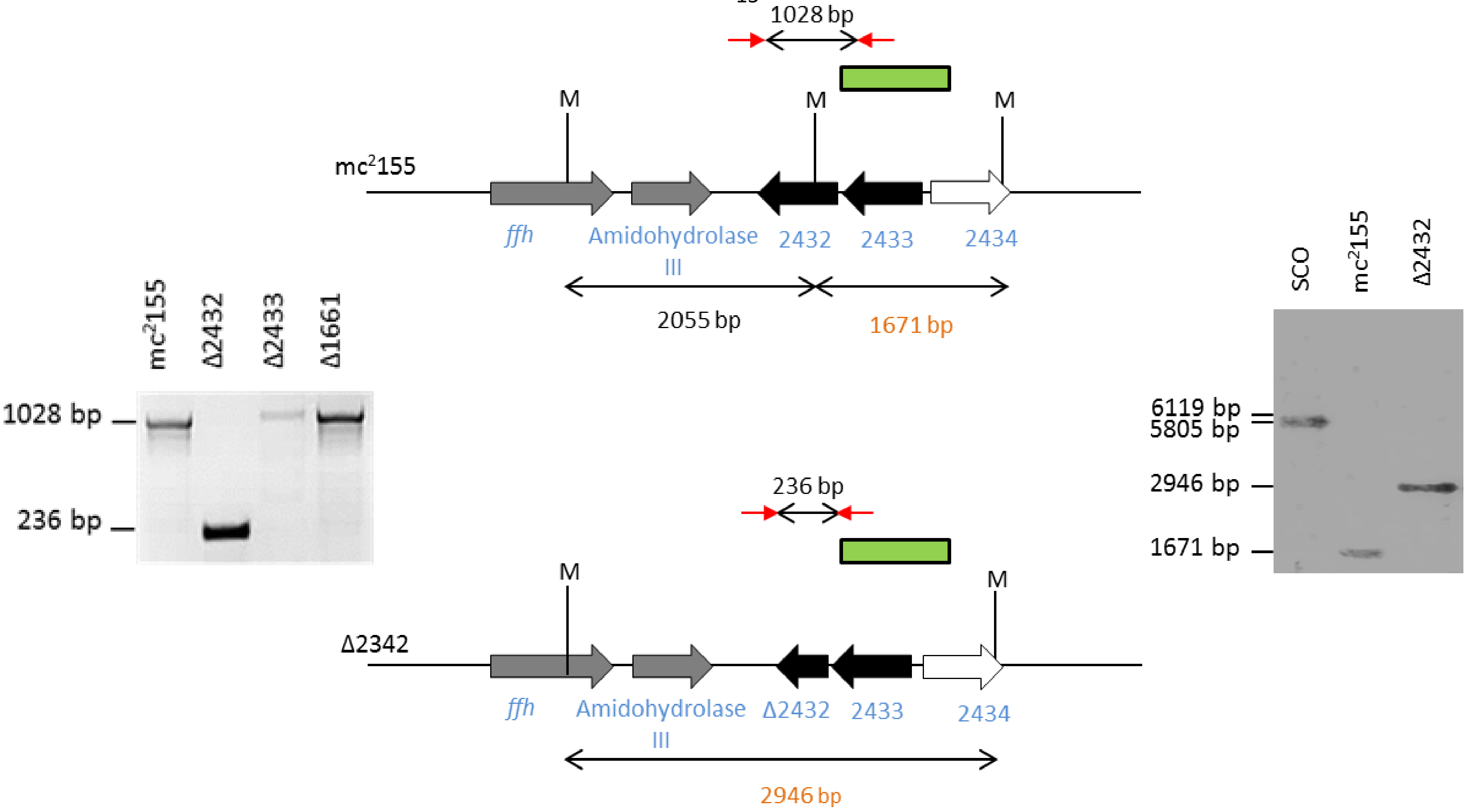

Figure S3A. Genotypic characterization of the MSMEG_2432 deletion mutant by PCR and Southern blot analysis. The genomic map of the relevant locus is shown for the wild-type and mutant strain. Also shown on the left is the PCR confirmation of the genotype and Southern Blotting is shown on the right. For PCR confirmation, chromosomal DNA was used to amplify the MSMEG_2432 alleles from the wild-type and mutant strains using the primer pairs described in Table S4 and indicated as red arrows above. The expected sizes of the amplicons are as follows: MSMEG_2432, 1028 bp and Δ2432, 236 bp. For the Southern blot analysis chromosomal DNA from the SCO, parental and mutant strain was digested with *Mlu*I [M]. The probe used for hybridization is shown as a solid green box and the expected sizes are indicated with orange text. The figures are not drawn to scale.

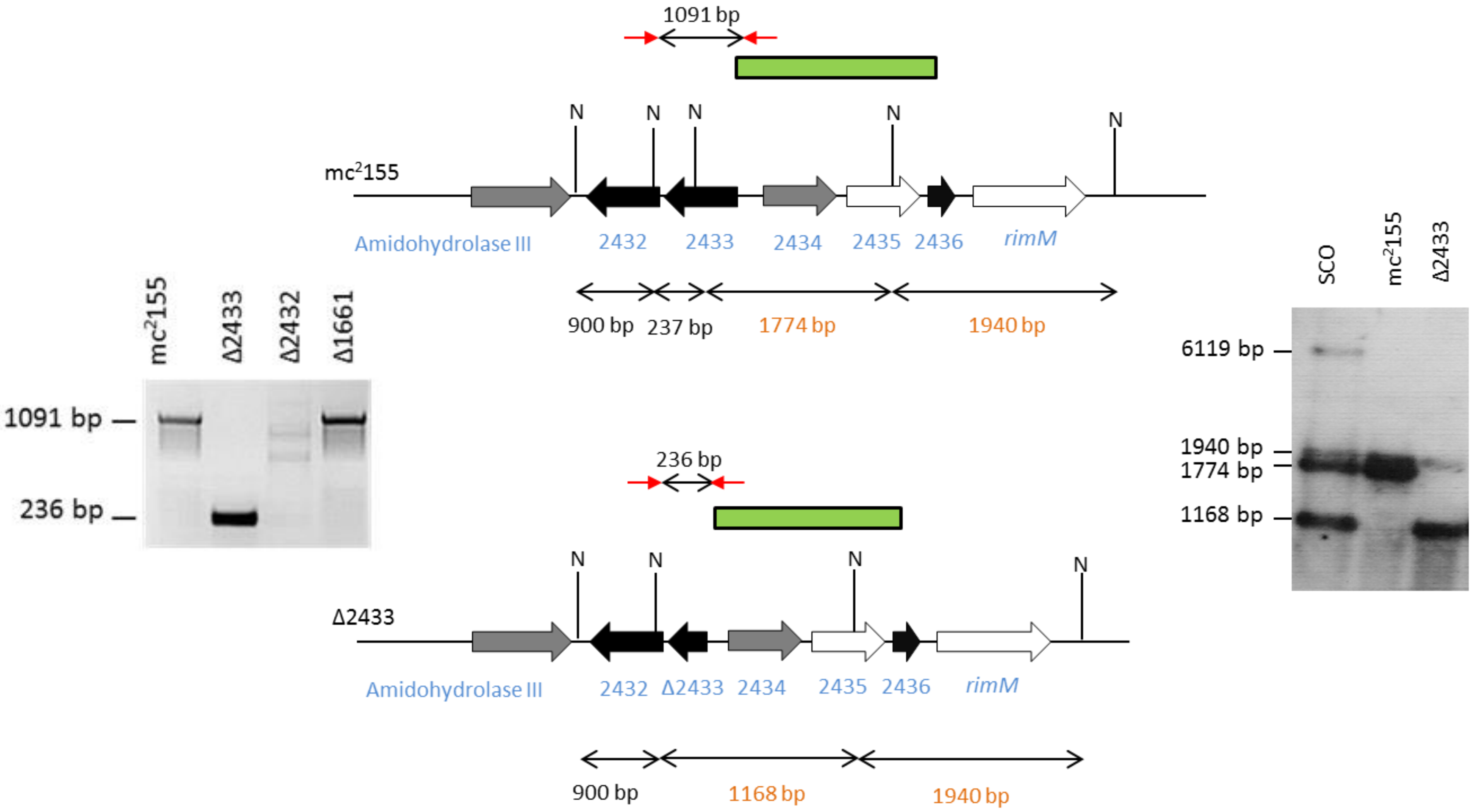

Figure S3B. Genotypic characterization of the MSMEG_2433 deletion mutant by PCR and Southern blot analysis. The genomic map of the relevant locus is shown for the wild-type and mutant strain. Also shown on the left is the PCR confirmation of the genotype and Southern Blotting is shown on the right. For PCR confirmation, chromosomal DNA was used to amplify the MSMEG_2433 alleles from the wild-type and mutant strains using the primer pairs described in Table S4 and indicated as red arrows above. The expected sizes of the amplicons are as follows: MSMEG_2433, 1091 bp and Δ2433, 236 bp. For the Southern blot analysis chromosomal DNA from the SCO, parental and mutant strain was digested with *Not*I [N]. The probe used for hybridization is shown as a solid green box and the expected sizes are indicated with orange text. The figures are not drawn to scale.

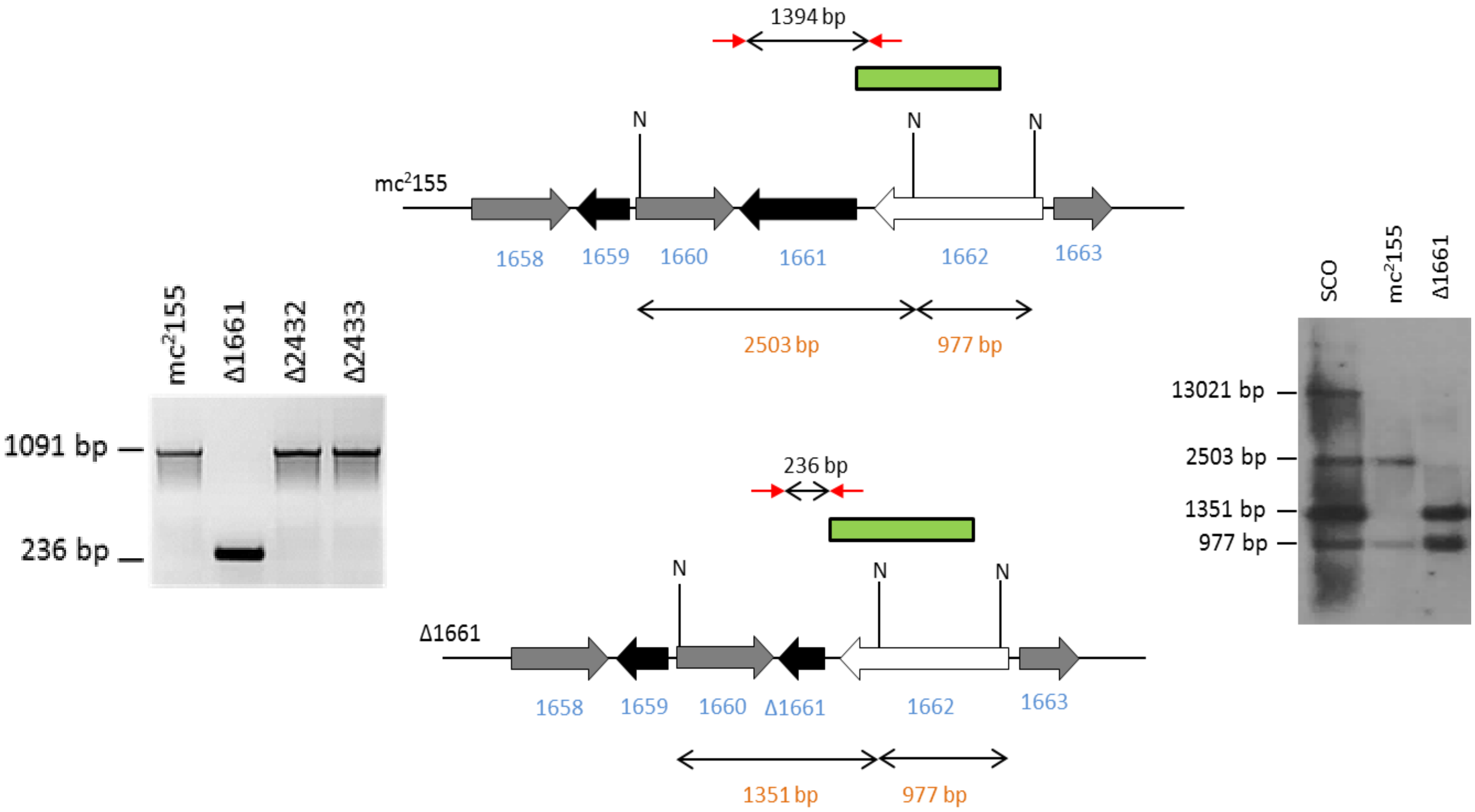

Figure S3C. Genotypic characterization of the MSMEG_1661 deletion mutant by PCR and Southern blot analysis. The genomic map of the relevant locus is shown for the wild-type and mutant strain. Also shown on the left is the PCR confirmation of the genotype and Southern Blotting is shown on the right. For PCR confirmation, chromosomal DNA was used to amplify the MSMEG_1661 alleles from the wild-type and mutant strains using the primer pairs described in Table S4 and indicated as red arrows above. The expected sizes of the amplicons are as follows: MSMEG_1661, 1394 bp and Δ1661, 236 bp. For the Southern blot analysis chromosomal DNA from the SCO, parental and mutant strain was digested with *Nco*I [N]. The probe used for hybridization is shown as a solid green box and the expected sizes are indicated with orange text. The figures are not drawn to scale.

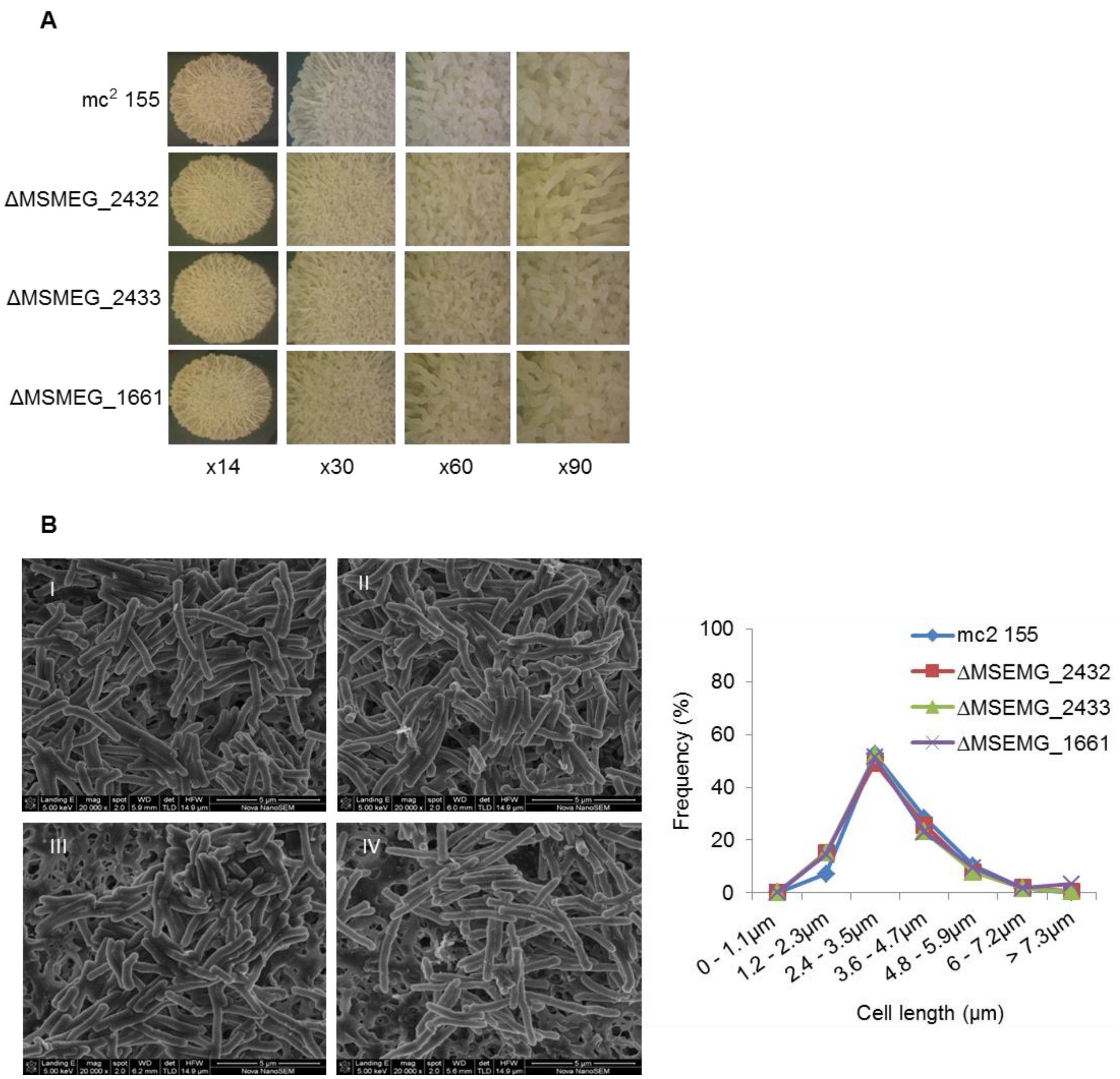

Figure S4. *In vitro* assessment of phenotypes in single deletion mutants. (A) Colony morphology of wild-type and single deletion mutants. Strains were grown to OD_{600nm} of 0.3 and 10 µl were subsequently spotted onto 7H10 solid media. Plates were incubated at 37 °C for 5 days and growth was monitored daily. (B) Representative SEM micrographs wild-type and mutant strains. Quantitative representation of distribution of cell lengths in wild-type and mutant strains. Number of cells counted for each strain (n) = 166, 203, 238 and 179, respectively.

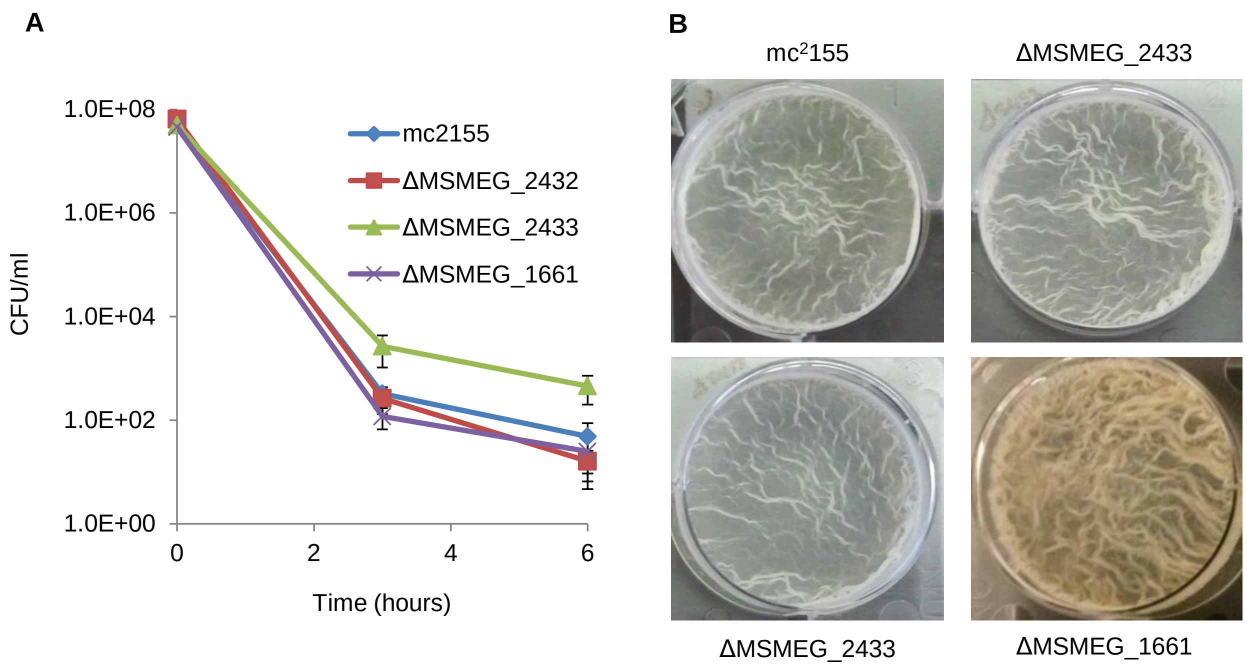

Figure S5. Assessment of changes in cell wall stability. (A) SDS killing (0.2%, w/v) of wild-type and single deletion mutant strains. Cultures were grown to OD_{600nm} of 0.3 following the addition of SDS at time 0. At 3 and 6 hours, 1 ml of sample was removed to prepare 10-fold serial dilutions for plating on 7H10 solid media. The data are the average of three independent experiments plotted with standard errors. (B) Assessment of biofilm formation in DD-CPase mutant strains. Biofilm formation was measured by the formation of a floating pellicle at the liquid-air interface in Sauton's minimal media (pH 7) at 37 °C for 5 days. Images are representative of three biological repeats.

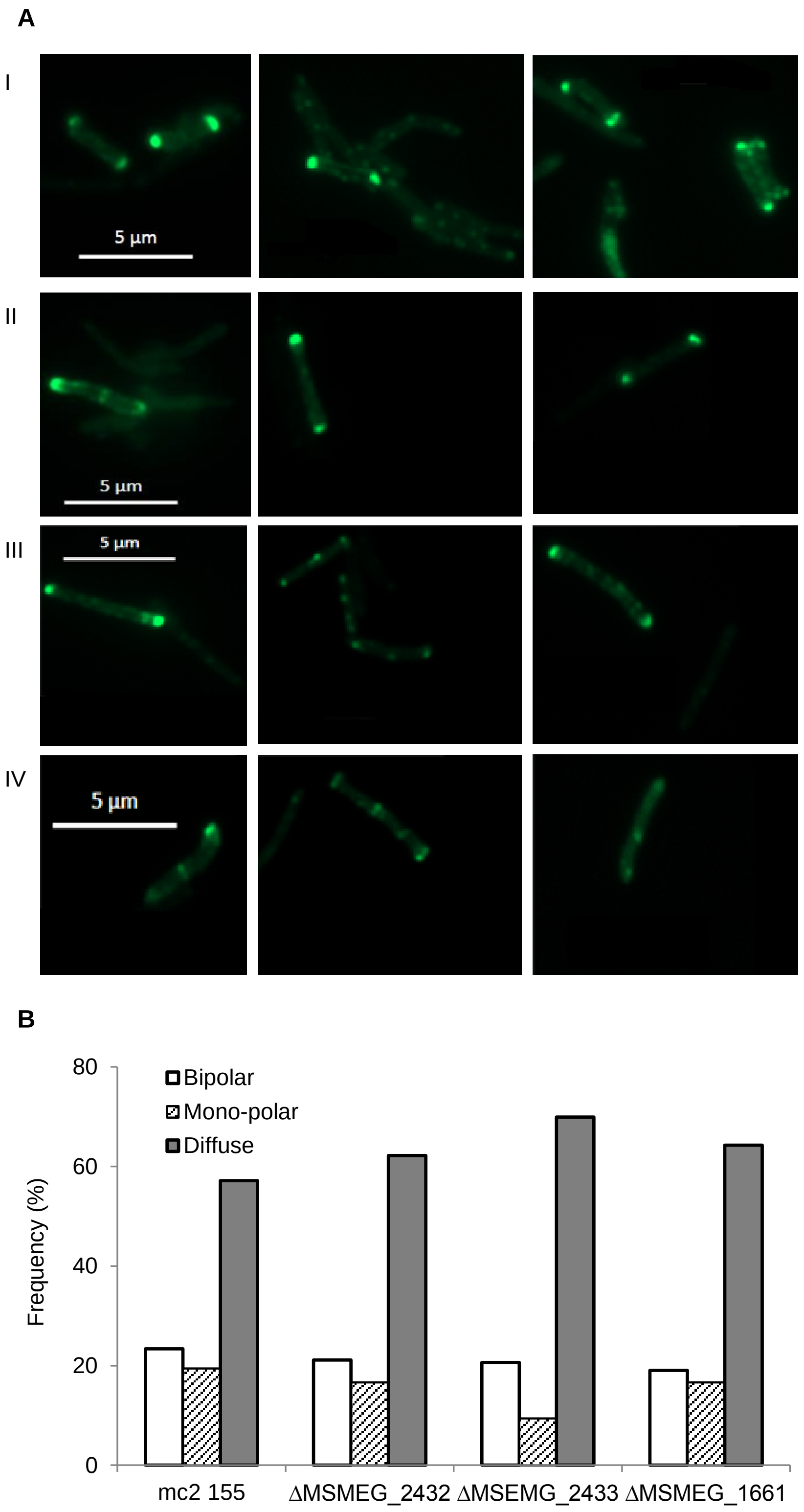

Figure S6. Fluorescence microscopy and quantitative assessment of peptidoglycan localization in DD-CPase deletion mutants. (A) BODIPY-vancomycin localization in *M. smegmatis*: (I) Wild-type, (II) Δ2432, (III) Δ2433, (IV) Δ1661 mutants. Strains were grown to OD_{600nm} of 0.3 and then stained with BODIPY-vancomycin, three representative images are shown for each strain. Scale bar = 5 µm. (B) Quantitative assessment of the distribution of staining patterns. Data is representative of one biological repeat where *n* (number of cells counted for each strain) = 77, 84, 90 and 223 for wild-type, ΔMSMEG_2432, ΔMSMEG_2433 and ΔMSMEG_1661 respectively.

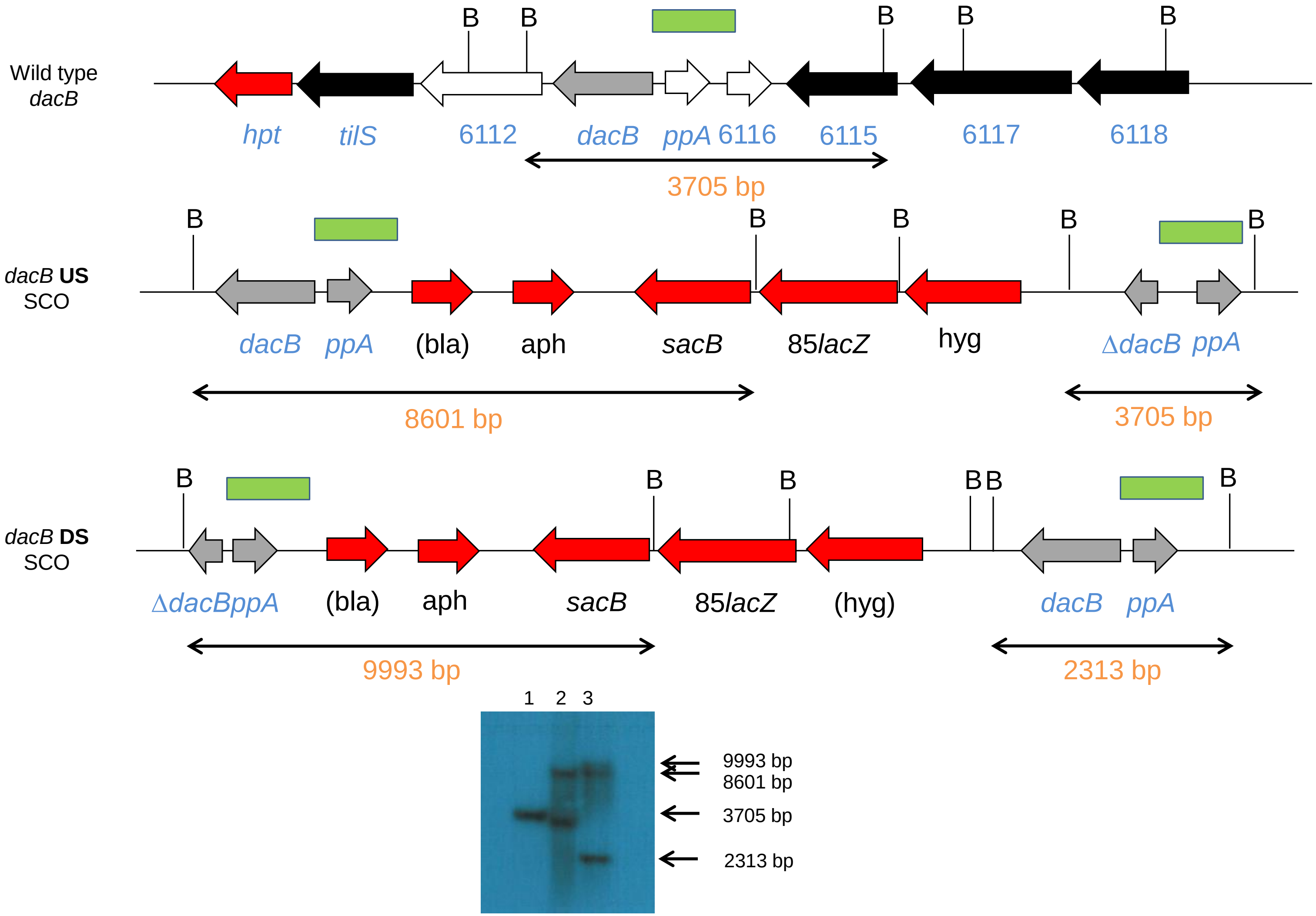

Figure S7A. Southern blot to confirm upstream and downstream SCOs for *dacB* mutant. Shown are the genomic maps for the wild-type, upstream (US) single crossover (SCO) and downstream (DS) SCO. Chromosomal DNA was isolated from wild-type (1), US SCO (2) and DS DSCO (3) and digested with *Bam*HI. The probe used for hybridization is shown as a solid green box and the expected sizes are indicated in orange text.

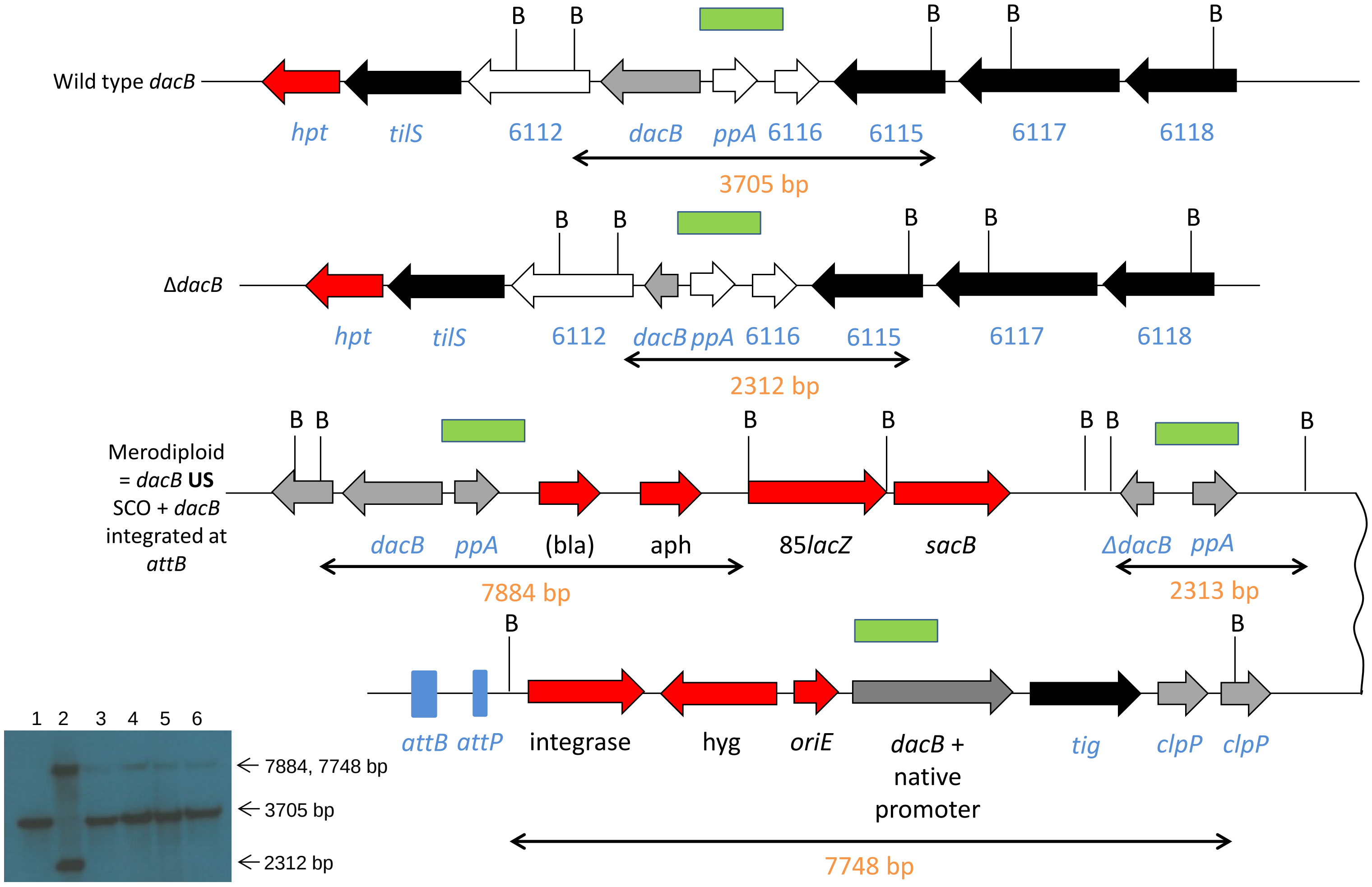

Figure S7B. Attempts to delete *dacB* in a merodiploid strain containing an additional copy of *dacB*, inserted at the *attB* site. Shown are genomic maps corresponding to wild-type, Δ*dacB*, the upstream single crossover (SCO) and resulting merodiploid strain. Southern hybridizations were performed by digesting 2 μg of chromosomal DNA isolated from the wild-type (1), the merodiploid strain (2) and possible double crossover recombinants isolated from counter selection of the merodiploid strain (3-6), was digested with *Bam*HI and probed with a region homologous to 1.2 kb upstream of *dacB* (green box). Expected fragment sizes are indicated in orange text. Counter selection yielded the wild-type (3705 bp) and not the mutant (2312 bp) band. Figure not drawn to scale.

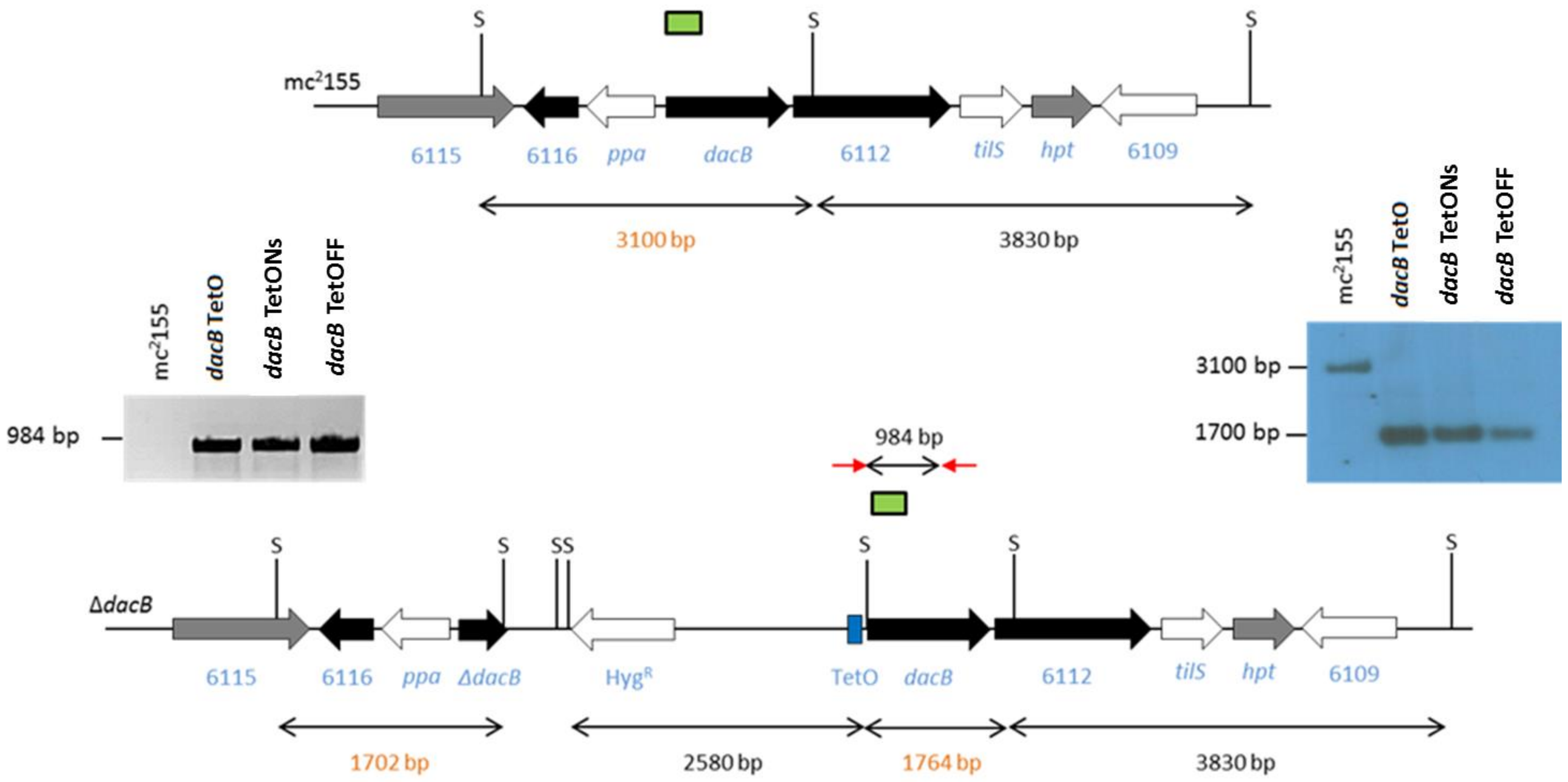

Figure S8. Genotypic characterization of the MSMEG_6113 (*dacB*) promoter replacement mutant by PCR and Southern blot analysis. The genomic map of the relevant locus is shown for the wild-type and mutant strain. Also shown on the left is the PCR confirmation of the genotype and Southern blotting is shown on the right. For PCR confirmation, chromosomal DNA was used to yield an amplicon specific for *dacB* directly downstream of Pmyc1tetO from the mutant strains (absent in wild-type) using the primer pairs described in Table S4, shown as red arrows. The expected sizes of all amplicons are 984 bp. For the Southern blot analysis chromosomal DNA from the parental and mutant strains were digested with *Sma*I [S]. The probe used for hybridization is shown as a solid green box and the expected sizes are indicated with orange text. The figures are not drawn to scale.

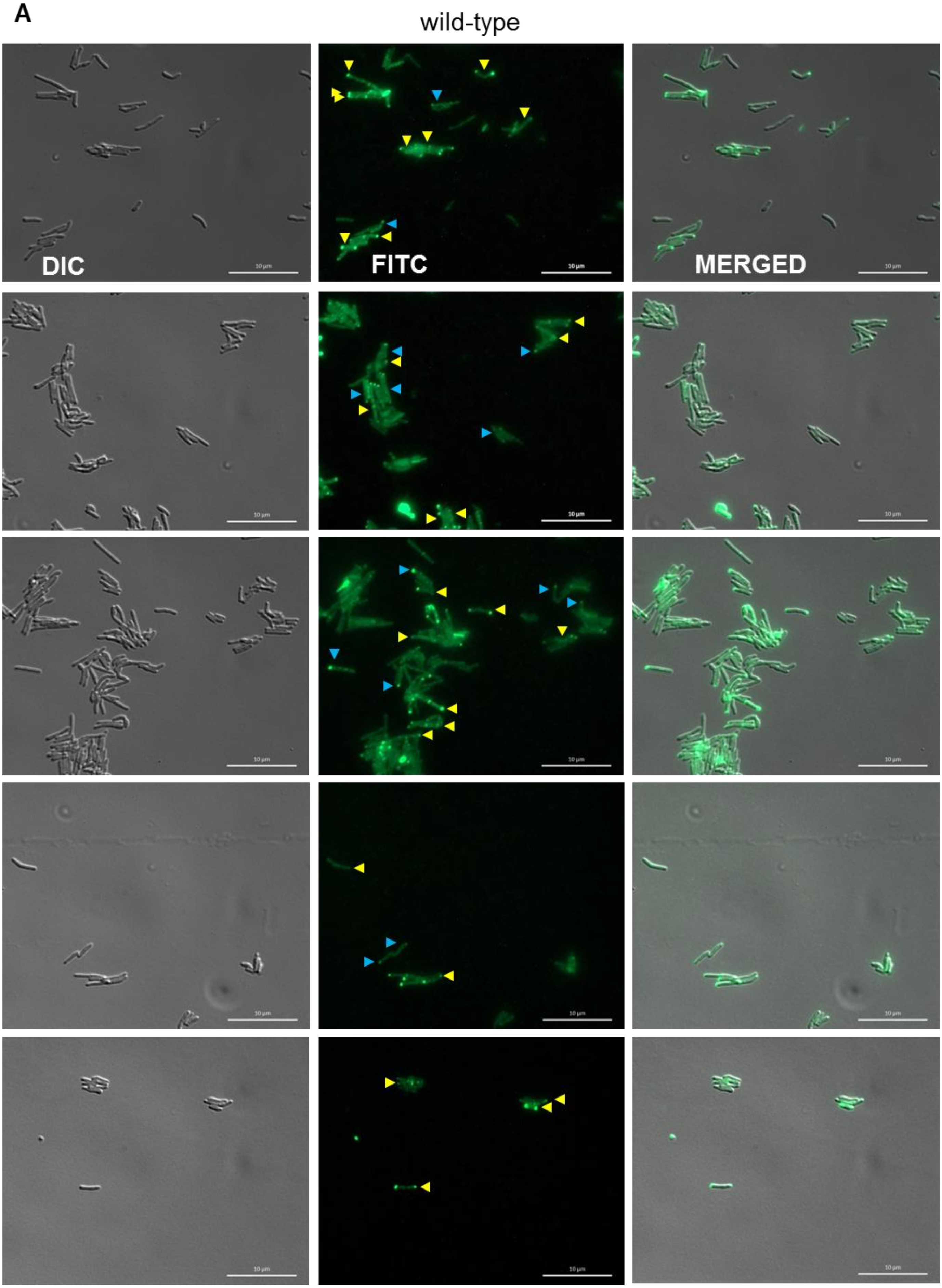
A
wild-type
DIC
FITC
MERGED
10 µm

wild-type

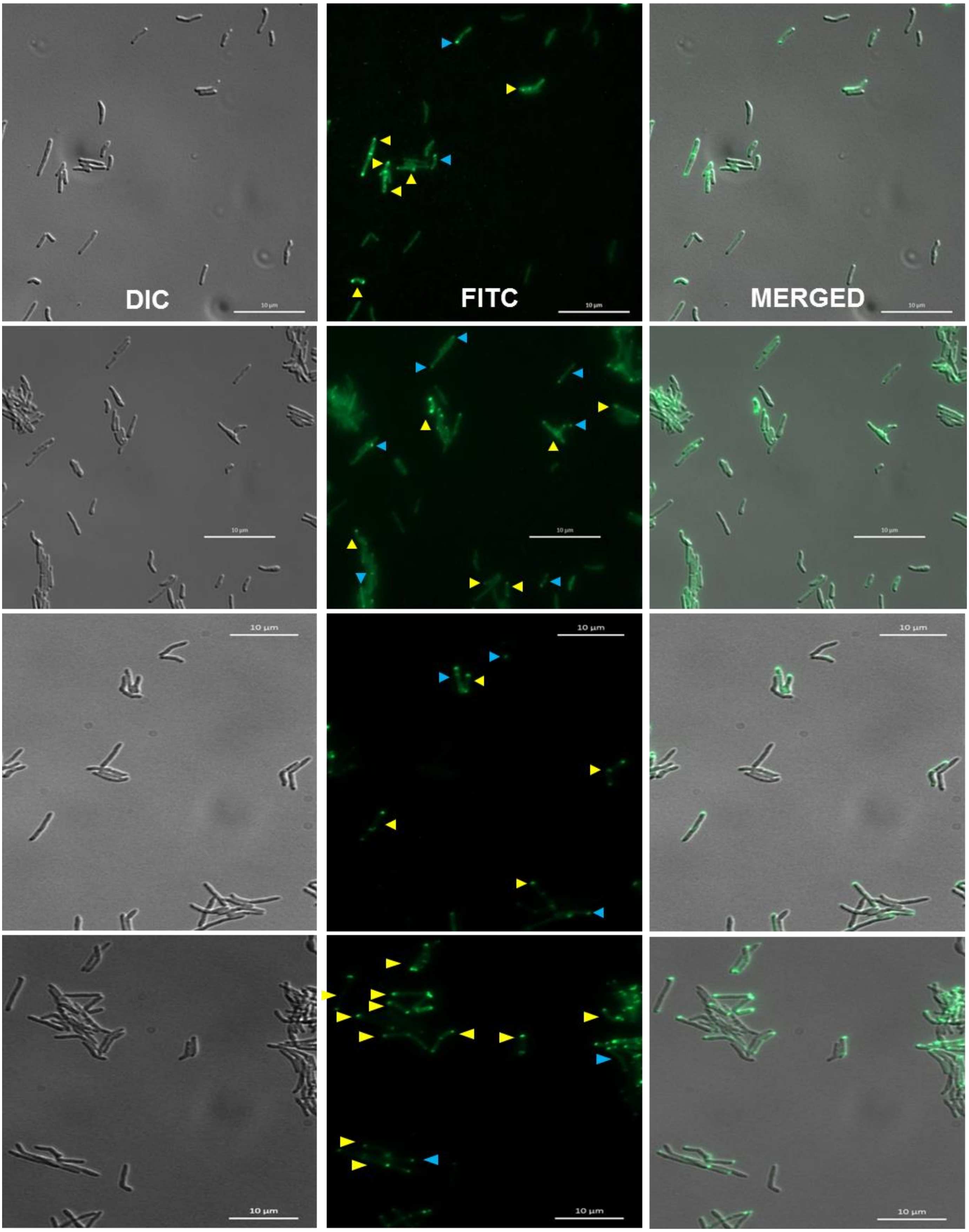

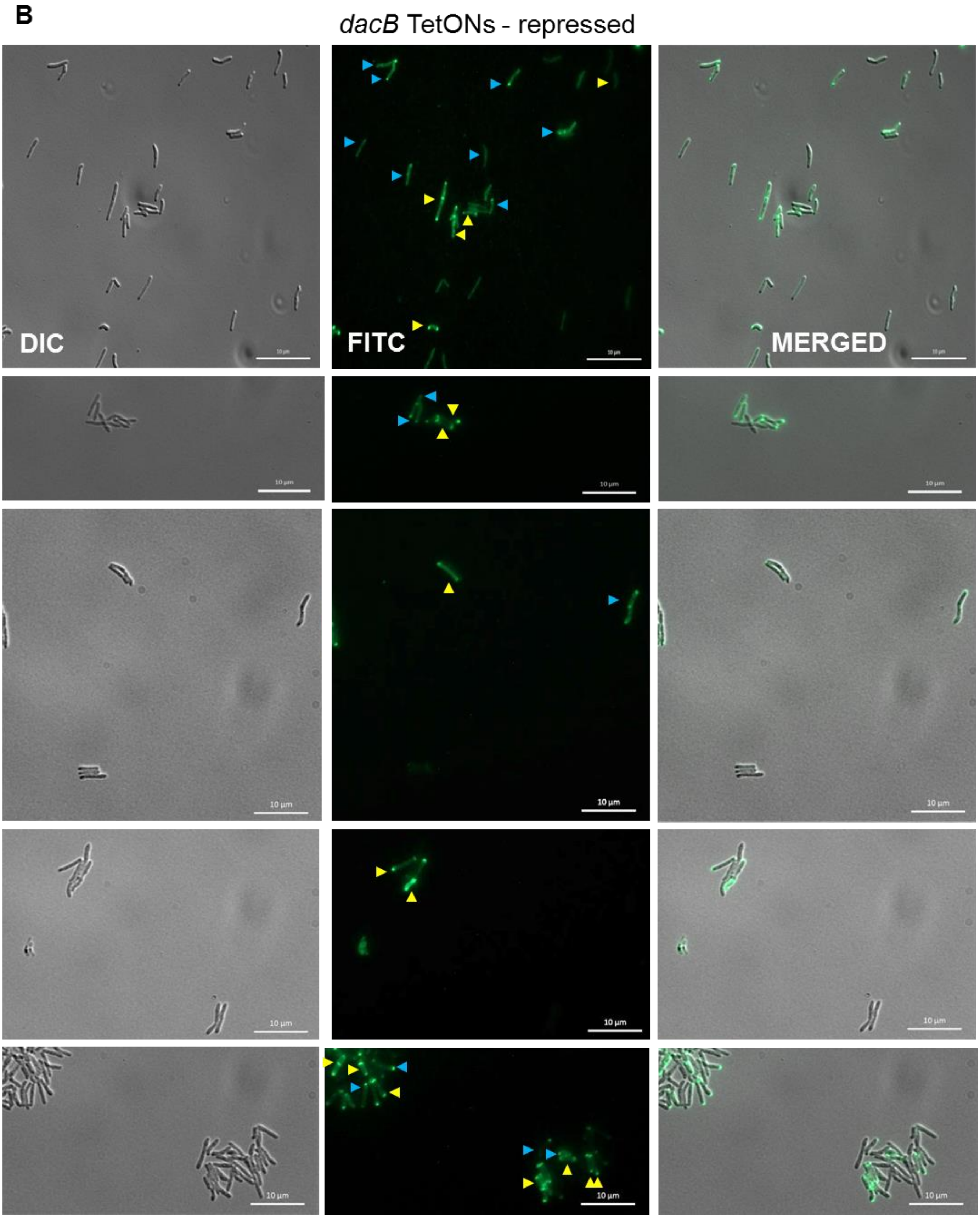
B
dacB TetONs - repressed
DIC
FITC
MERGED
10 μm

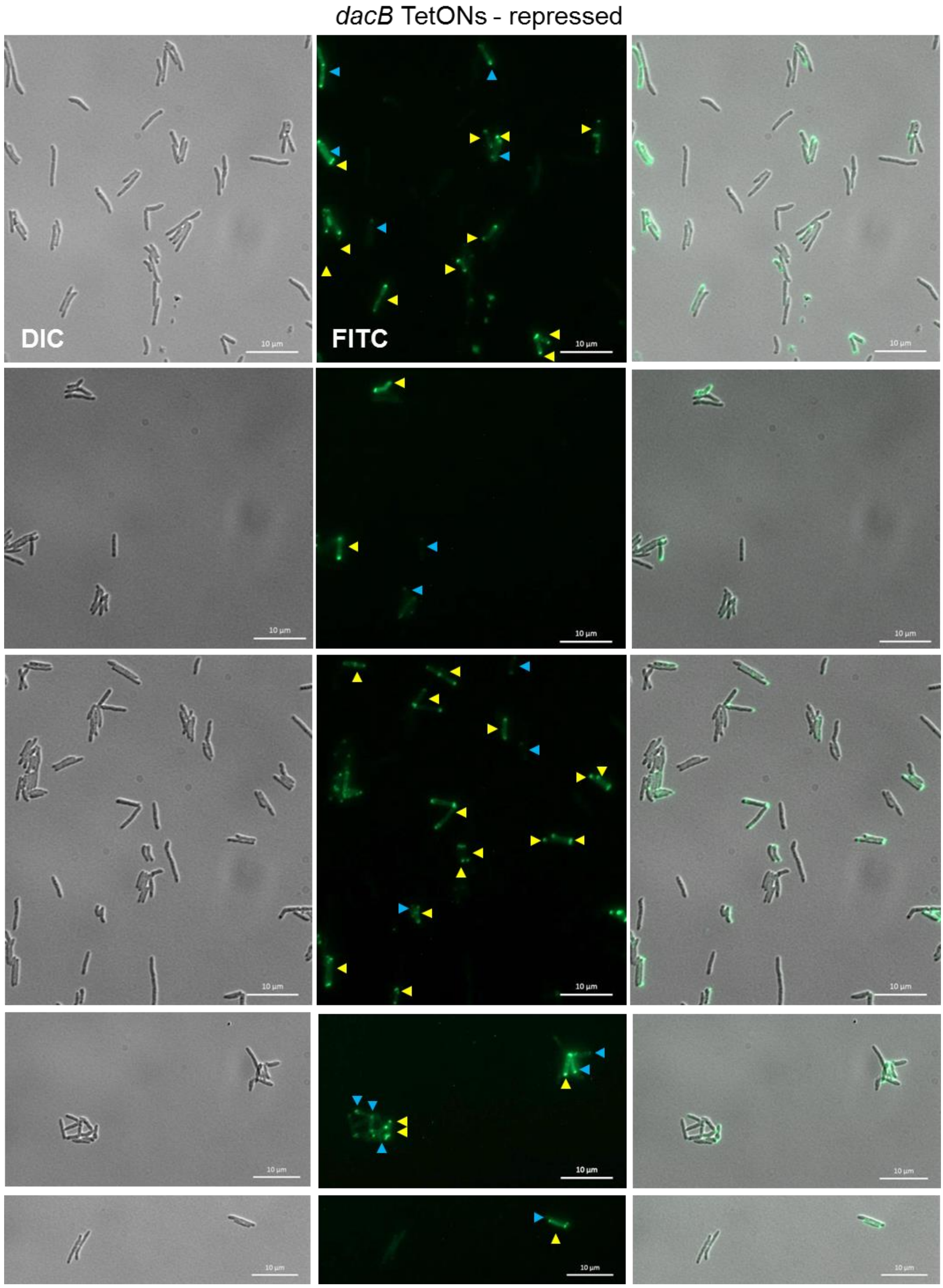
dacB TetONs - repressed
DIC
FITC
10 µm

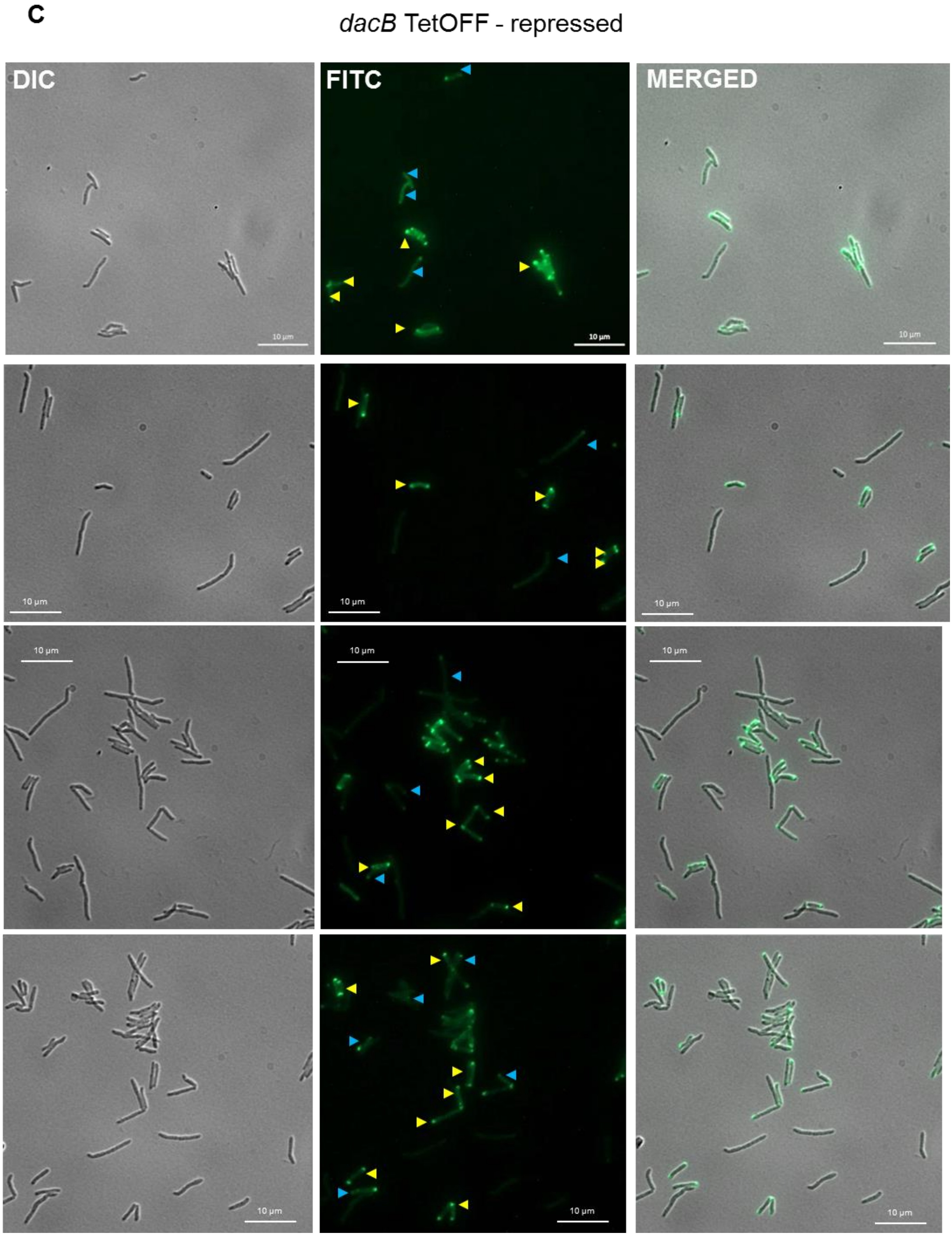
C
dacB TetOFF - repressed
DIC
FITC
MERGED
10 µm

dacB TetOFF – repressed

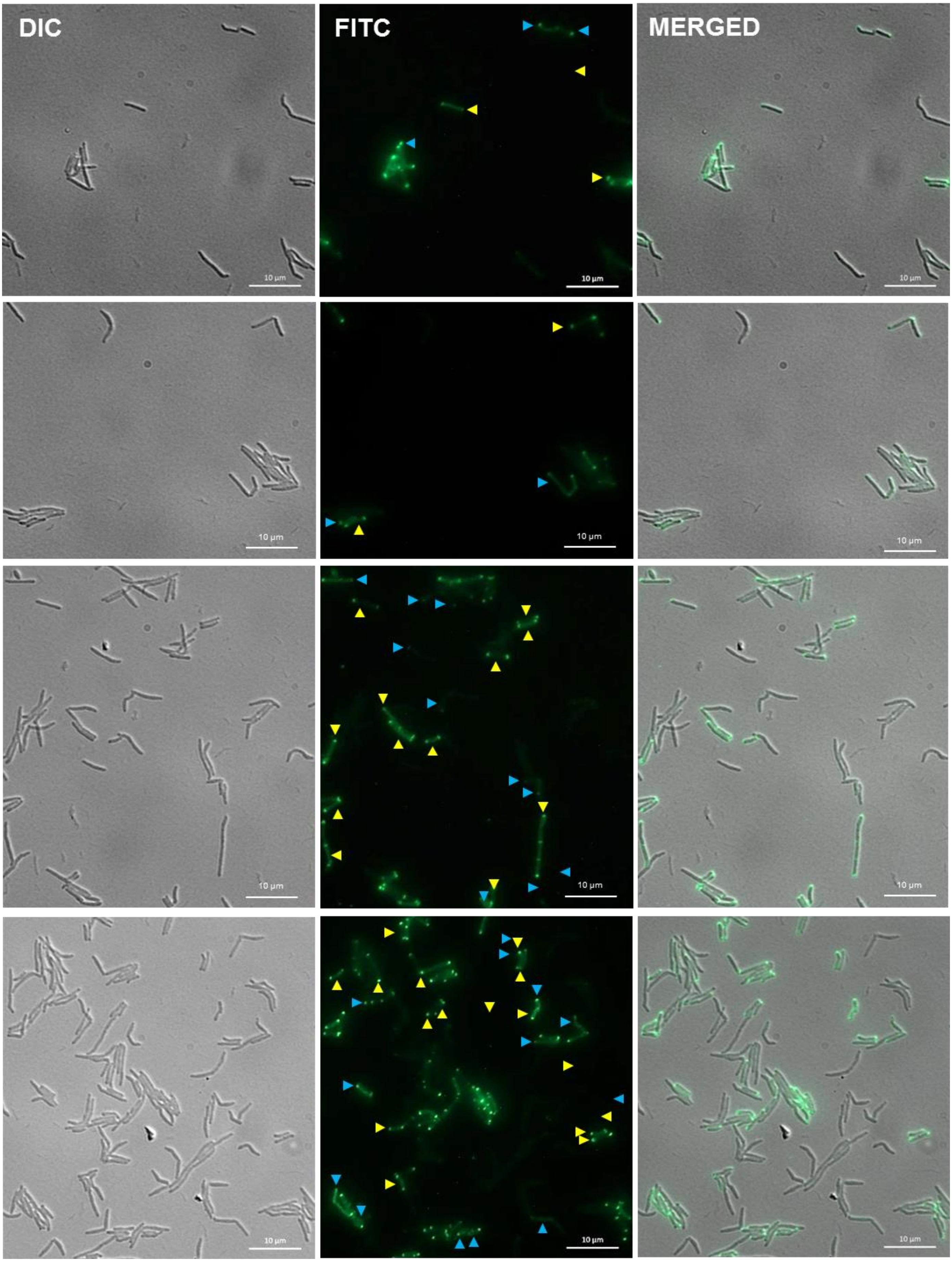

Figure S9. Representative images of PG staining patterns observed in *dacB* knockdown strains. Cells corresponding to the wild-type (A), *dacB* TetON$_s$ 0 ng/ml aTc (repressed) (B) and *dacB* TetOFF 100 ng/ml aTc (repressed) (C) were stained with BODIPY-vancomycin during late logarithmic growth. Images were captured in the DIC and FITC channels and staining patterns were quantitated as either bipolar (yellow arrows) or monopolar (blue arrows). Scale bars depicted in each image.

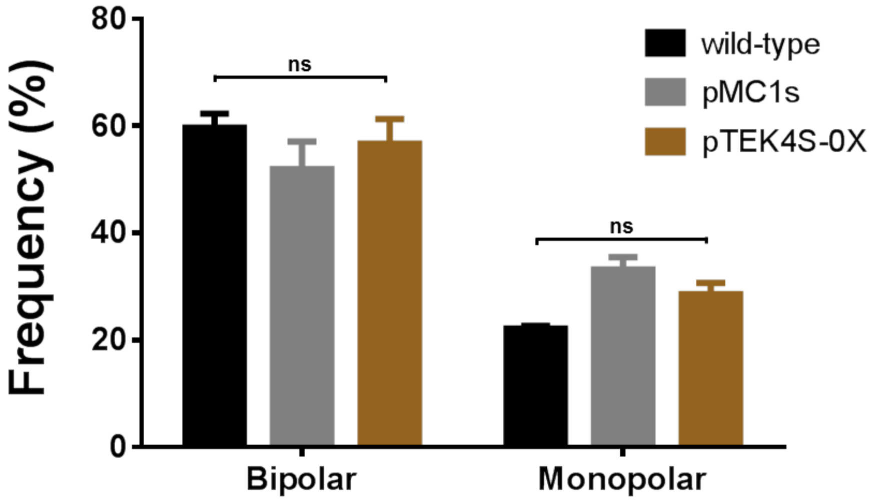

Figure S10. BODIPY-vancomycin staining of wild-type and repressor only (pMC1s or pTEK4S-0X) strains. Strains were sampled during late logarithmic growth and stained. Staining patterns were viewed under bright-field (DIC) and FITC-channels and scored as bipolar or monopolar. Data are represented as the average of three independent biological repeats with error bars depicting standard error from the mean. No statistically (ns) significant differences were detected, comparing strains in each staining type.

References

1. Snapper, S., Melton, R., Mustafa, S., Kieser, T. & Jacobs Jr., W. Isolation and characterization of efficient plasmid transformation mutants of Mycobacterium smegmatis. *Mol Microbiol* **4,** 1911–9 (1990).

2. Parish, T. & Stoker, N. Use of a flexible cassette method to generate a double unmarked Mycobacterium tuberculosis tlyA plcABC mutant by gene replacement. *Microbiology* **146,** 1969–75 (2000).
